# Supplementary material for: A KRAS-responsive long non-coding RNA controls microRNA processing
Source: Nat Commun. 2021 Apr 1;12:2038. doi: 10.1038/s41467-021-22337-3 (PMC8016872; doi:10.1038/s41467-021-22337-3)
Supplement: Supplementary file 1 — Supplementary Information [file 41467_2021_22337_MOESM1_ESM.pdf]

a

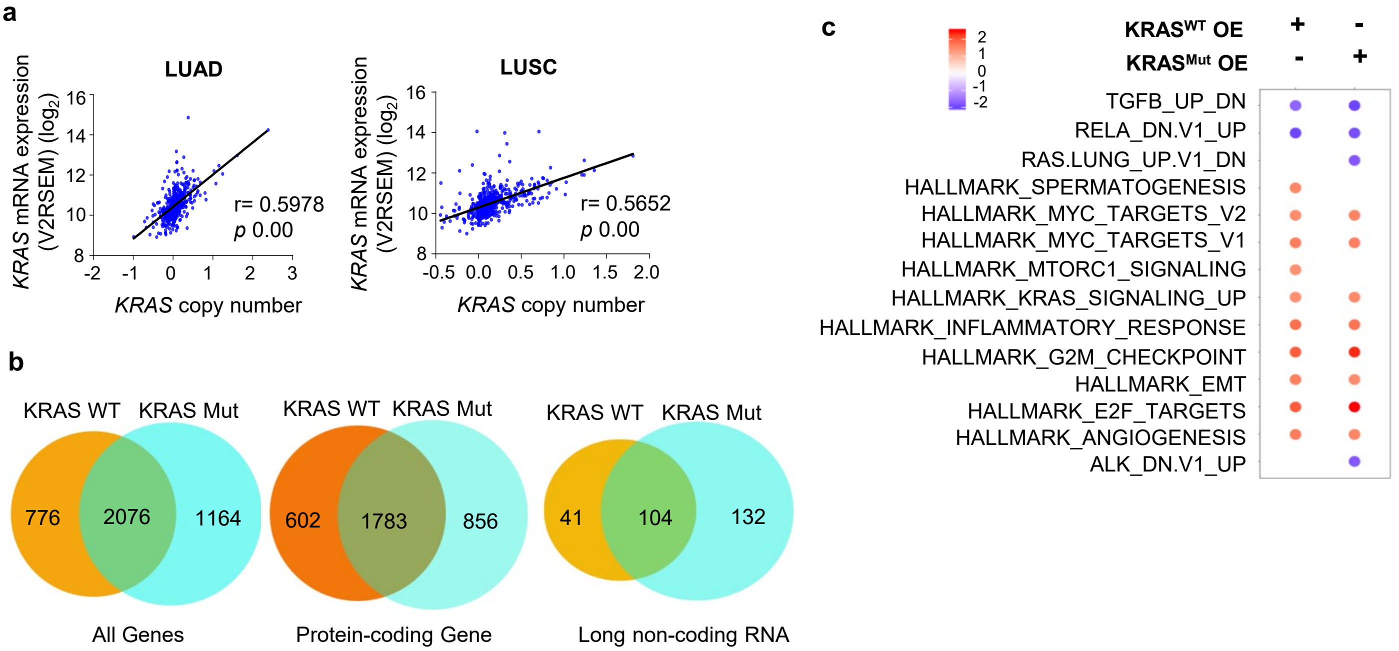

d

Gene	LINC02575 (ENSG00000228709)
Description	Long intergenic non-protein coding RNA 2575
Gene Synonyms	AP001065.15
Location	Chromosome 21: 44,485,577-44,490,288, forward strand GRCh38:CM000683.2
About this gene	This gene has 1 transcript
Transcription ID	ENST00000449713.1
Size (bp)	912

e

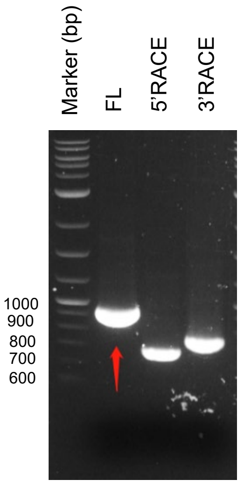

f

Locus conservation	KIMAT1
Chimp	No
Mouse	No
Fish	No
Fly	No

g

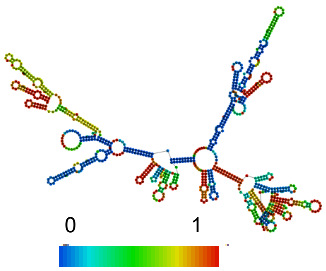

h

Gene name	Ensemble ID	Coding probability	Coding capacity
KIMAT1	ENSG00000228709	0.02	No
KRAS	ENSG00000133703	0.79	Yes
MALAT1	ENSG00000251562	0.04	No

i

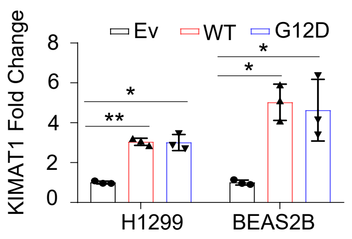

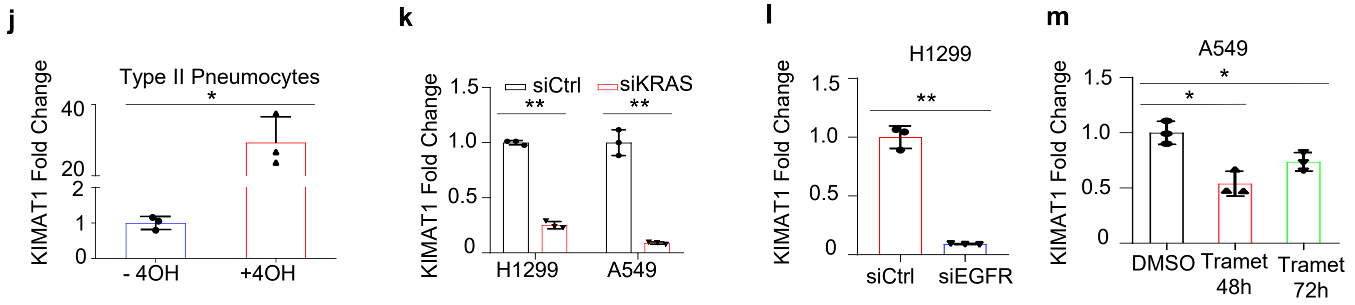

Supplementary Figure 1: *KIMAT1* is a downstream effector of KRAS. a, *KRAS* mRNA expression positively correlates with *KRAS* DNA copy number in LUAD and LUSC datasets (TCGA). On the x axis is reported the SNP-array-inferred *KRAS* copy number, on the y axis is reported *KRAS* mRNA expression by RNA-seq set to $\log_2$ scale. **b**, Number of genes in common between $KRAS^{WT}$ and $KRAS^{Mut}$. **c,** GSEA analysis of pathways commonly modulated by $KRAS^{WT}$ and $KRAS^{Mut}$. The heatmap indicates the GSEA scores. **d**, *KIMAT1* isoform and location (Gene official symbol linc02575) from Ensembl. **e**, *KIMAT1* full length was determined by 5' (lane 2) and 3' (lane 3) rapid-amplification of cDNA ends (RACE) assays. *KIMAT1* total length is 912 nucleotides (lane 1, red arrow). Data are representative of 2 independent experiments. **f**, As annotated in LNCipedia, *KIMAT1* is not conserved in other species. **g**, RNAfold prediction of base-pairing probabilities of *KIMAT1* secondary structure with a minimum free energy (MFE) of -337.53 kcal/mol indicating that the structure is in thermodynamic ensemble. The colour scale indicates the confidence of the prediction for each base. **h**, Coding probability scores for *KIMAT1* were assessed by Coding Potential Assessment Tool (CPAT). *KRAS* and *MALAT1* were used as positive and negative controls, respectively. **i**, qRT-PCR for *KIMAT1* 48h after transfection with $KRAS^{WT}$ or $KRAS^{G12D}$ in H1299 or BEAS2B cells. H1299 **= 5.23E-05, *= 0.0010; BEAS2B *= 0.0016 and 0.01548 from left to right. **j**, qRT-PCR for *KIMAT1* in Type II Pneumocytes $KRAS^{G12V}$-inducible cells after treatment with 4-Hydroxytamoxifen (4OH) to activate KRAS. *= 0.002489. **k**, qRT-PCR for *KIMAT1* upon KRAS silencing in H1299 and A549 cells. **= 4.73E-06 (H1299) and 0.00018 (A549). **l**, Downregulation of *KIMAT1* determined by qRT-PCR in cells transfected with a pool of 4 siRNA targeting the EGFR compared to cells transfected with a non-targeting siRNA (siCtrl). **= 8.05E-05. **m**, qRT-PCR for *KIMAT1* in A549 cells after treatment with trametinib for 48h or 72h. *= 0.006478 (Tramet 48h vs Ctrl) and 0.02732 (Tramet 72h vs Ctrl). Error bars represent mean ± S.D (n=3) in panels with error bars. ***p* values were calculated by two-tailed Student's *t* test.

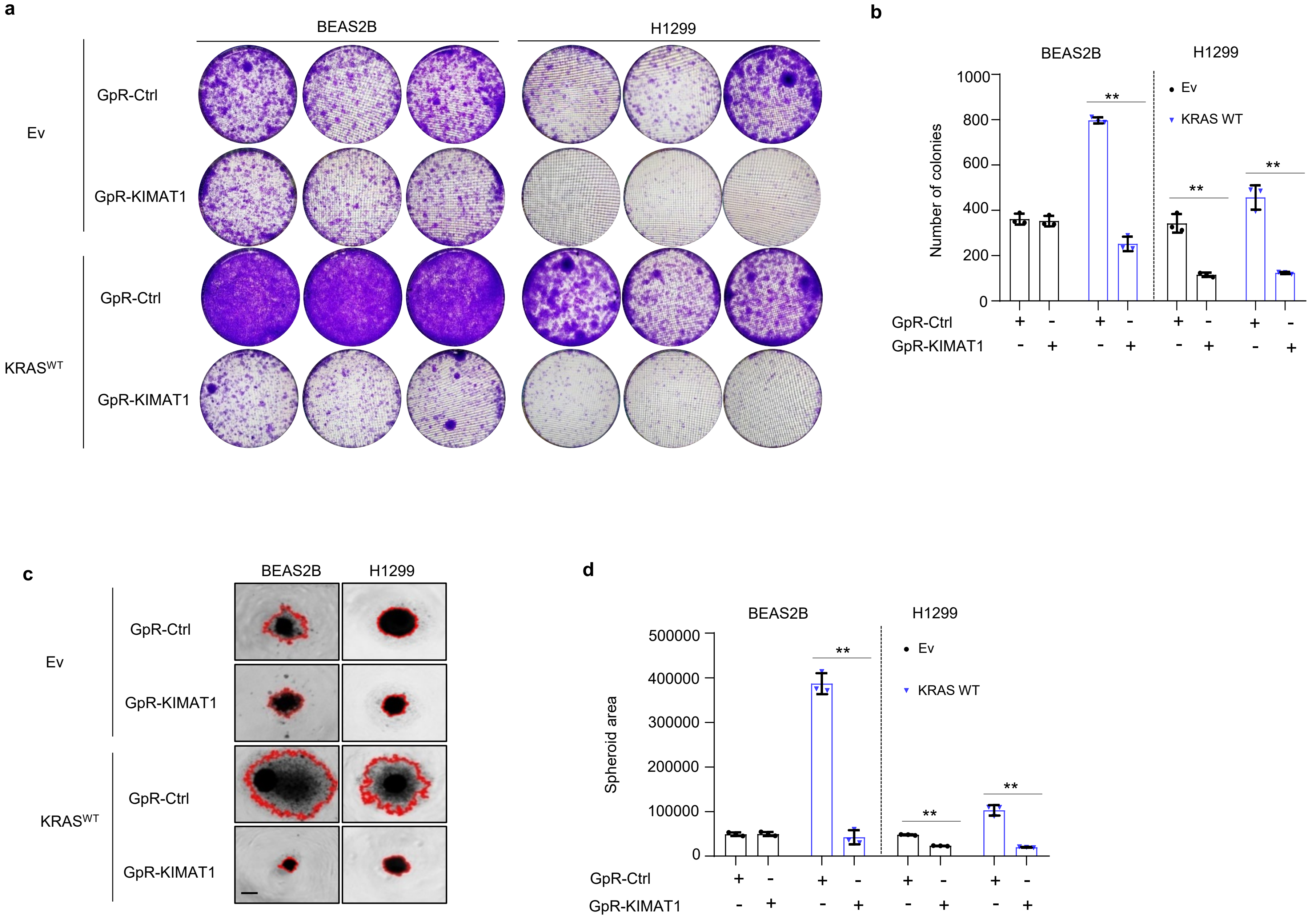
a
BEAS2B
H1299
Ev
GpR-Ctrl
GpR-KIMAT1
KRAS^WT
GpR-Ctrl
GpR-KIMAT1
b
BEAS2B
H1299
Number of colonies
1000
800
600
400
200
0
Ev
KRAS WT
**
GpR-Ctrl
GpR-KIMAT1
c
BEAS2B
H1299
Ev
GpR-Ctrl
GpR-KIMAT1
KRAS^WT
GpR-Ctrl
GpR-KIMAT1
d
BEAS2B
H1299
Spheroid area
500000
400000
300000
200000
100000
0
Ev
KRAS WT
**
GpR-Ctrl
GpR-KIMAT1

Supplementary Figure 2: *KIMAT1* is a downstream effector of KRAS.

a-d, Colony formation (a,b) and 3D cell invasion (c,d) assays and corresponding quantification in BEAS2B and H1299 cells stably expressing $KRAS^{WT}$ upon silencing of *KIMAT1.* Scale bar, 500 µm. Error bars represent mean ± S.D (n=3) in panels with error bars. *p* values from left to right **= 1.11E-05, 0.000735, 0.000423 (b); 3.04E-05, 6.49E-06, 0.00025 (d).by two-tailed Student's *t* test.

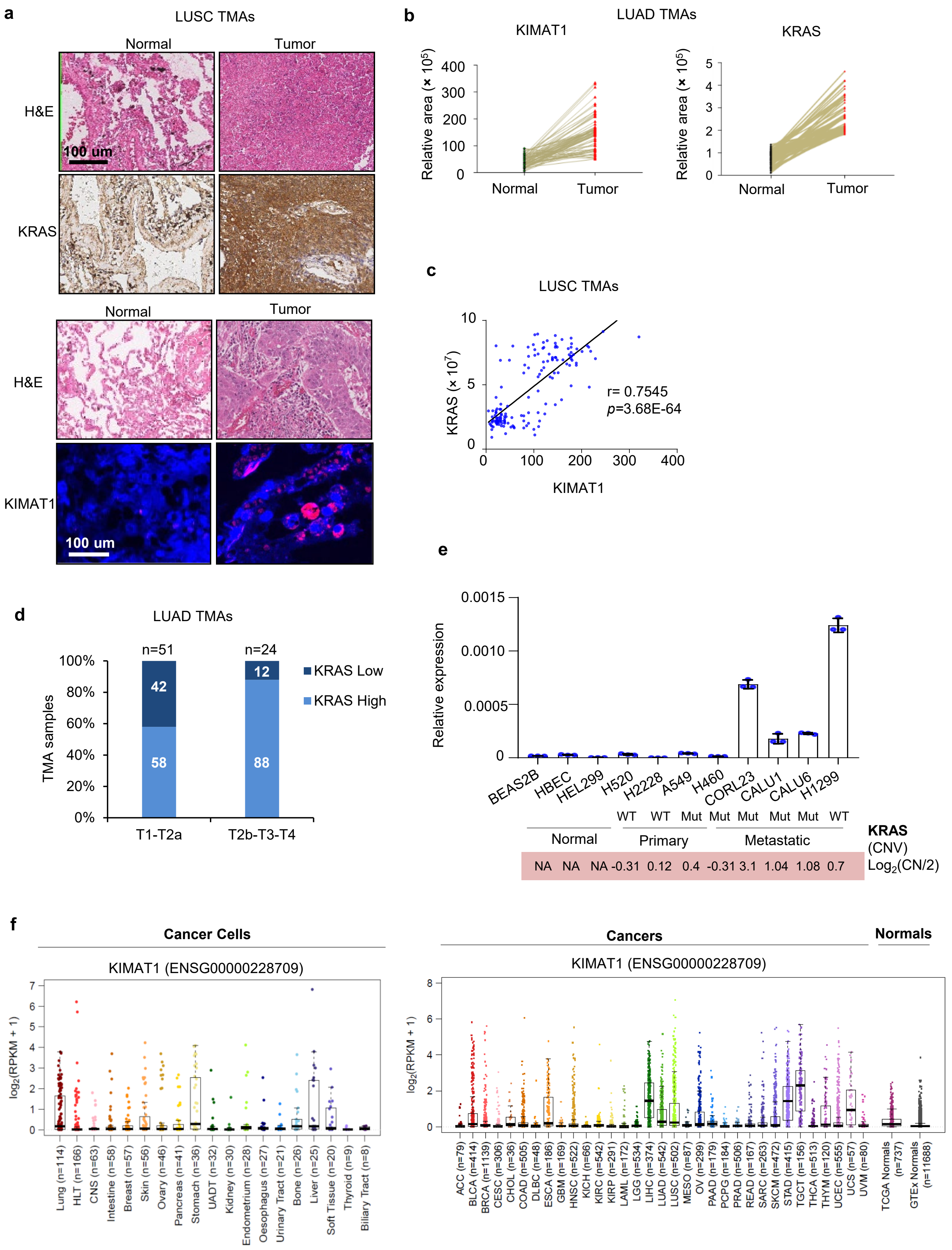
a
LUSC TMAs
Normal
Tumor
H&E
KRAS
100 um
H&E
KIMAT1
100 um
b
LUAD TMAs
KIMAT1
KRAS
Relative area (× 10^5)
Normal
Tumor
c
LUSC TMAs
KRAS (× 10^7)
KIMAT1
r= 0.7545
p=3.68E-64
d
LUAD TMAs
n=51
n=24
KRAS Low
KRAS High
TMA samples
T1-T2a
T2b-T3-T4
42
58
12
88
e
Relative expression
BEAS2B
HBEC
HEL299
H520
H2228
A549
H460
CORL23
CALU1
CALU6
H1299
WT WT Mut Mut Mut Mut Mut WT
Normal
Primary
Metastatic
KRAS
(CNV)
Log2(CN/2)
NA NA NA -0.31 0.12 0.4 -0.31 3.1 1.04 1.08 0.7
f
Cancer Cells
KIMAT1 (ENSG00000228709)
log2(RPKM + 1)
Lung (n=114)
HLT (n=166)
CNS (n=63)
Intestine (n=58)
Breast (n=57)
Skin (n=56)
Ovary (n=46)
Pancreas (n=41)
Stomach (n=36)
UADT (n=32)
Kidney (n=30)
Endometrium (n=28)
Oesophagus (n=27)
Urinary Tract (n=21)
Bone (n=26)
Liver (n=25)
Soft Tissue (n=20)
Thyroid (n=9)
Biliary Tract (n=8)
Cancers
Normals
KIMAT1 (ENSG00000228709)
ACC (n=79)
BLCA (n=414)
BRCA (n=1139)
CESC (n=306)
CHOL (n=36)
COAD (n=505)
DLBC (n=48)
ESCA (n=186)
GBM (n=169)
HNSC (n=522)
KICH (n=66)
KIRC (n=542)
KIRP (n=291)
LAML (n=172)
LGG (n=534)
LIHC (n=374)
LUAD (n=542)
LUSC (n=502)
MESO (n=87)
OV (n=299)
PAAD (n=179)
PCPG (n=184)
PRAD (n=506)
READ (n=167)
SARC (n=263)
SKCM (n=472)
STAD (n=415)
TGCT (n=156)
THCA (n=513)
THYM (n=120)
UCEC (n=555)
UCS (n=57)
UVM (n=80)
TCGA Normals (n=737)
GTEx Normals (n=11688)

Supplementary Figure 3: *KIMAT1* is overexpressed in lung squamous cell carcinoma and other cancer types. a,**b** IHC for KRAS, smRNA FISH for *KIMAT1* and corresponding quantification of KRAS and *KIMAT1* expression levels in 75 FFPE LUSC lesions compared to 75 matched normal lung samples. Spots were counted using the online JAVA software from StarSearch. **c**, Pearson correlation between *KIMAT1 and KRAS* in matched normal/tumor LUSC samples. *p* values were calculated by two-tailed Student's *t* test. **d**, KRAS expression (%) in early-stage versus late-stage tumors shown in Fig. 1e. **e**, qPCR for *KIMAT1* expression in a panel of normal, primary and metastatic lung adenocarcinoma and squamous cell carcinoma cell lines. *KRAS* copy number (log_2CN/2) for the indicated cells reported in the table was downloaded from CCLE data portal. Mean ± S.D (n=3). **f**, (Left) *KIMAT1* expression in 863 different cancer cell lines from CCLE. HLT: Haematopoietic and Lymphoid Tissue; CNS: Central Nervous System; UADT: Upper Aerodigestive Tract. (Right) *KIMAT1* expression in 33 TCGA tumor types (n=10480), NAT (Normal adjacent to tumors, n=737) and GTEx normal tissue RNAseq datasets (n=11688). See Source File.

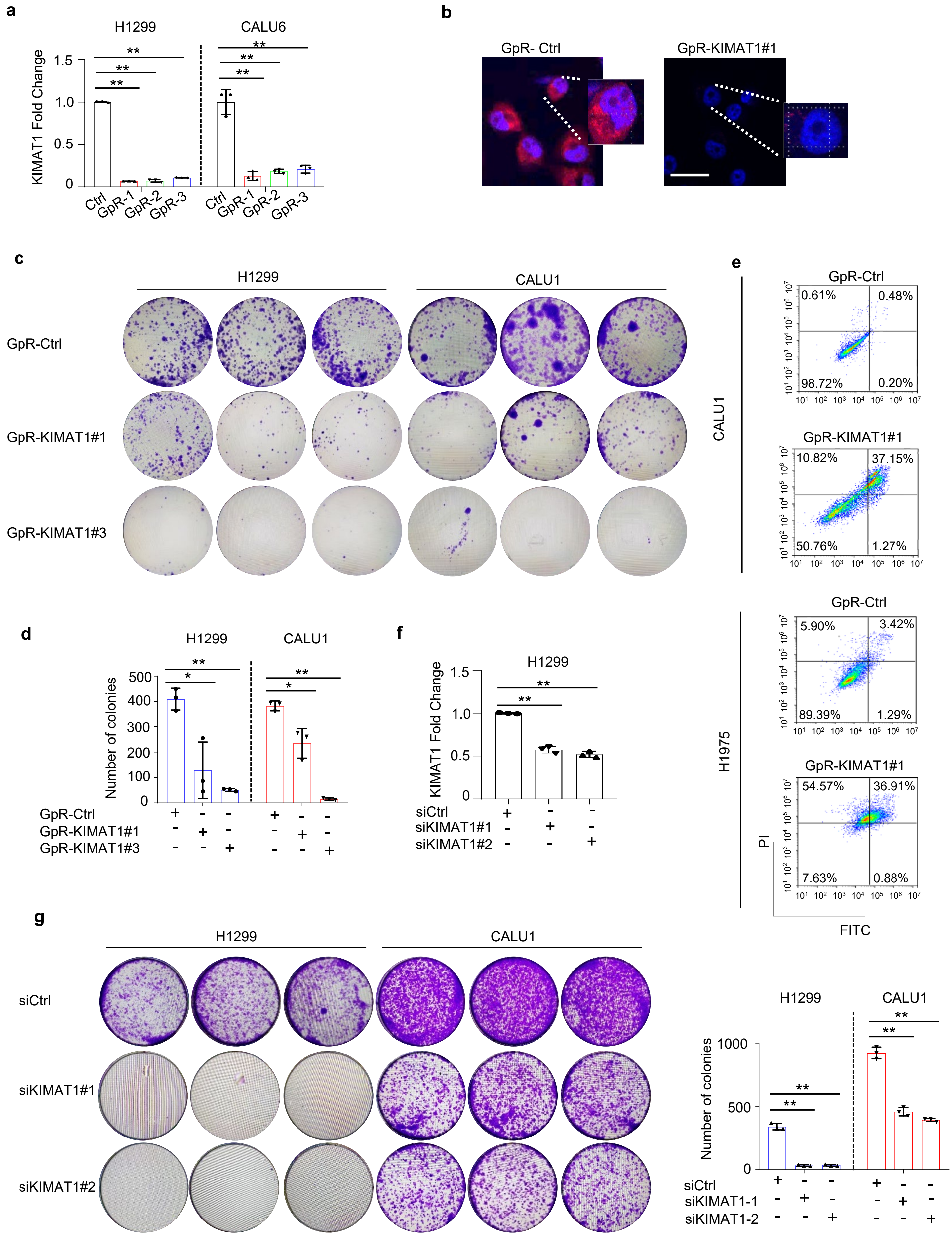
a
H1299
CALU6
KIMAT1 Fold Change
Ctrl
GpR-1
GpR-2
GpR-3
b
GpR- Ctrl
GpR-KIMAT1#1
c
H1299
CALU1
GpR-Ctrl
GpR-KIMAT1#1
GpR-KIMAT1#3
e
CALU1
GpR-Ctrl
0.61%
0.48%
98.72%
0.20%
GpR-KIMAT1#1
10.82%
37.15%
50.76%
1.27%
H1975
GpR-Ctrl
5.90%
3.42%
89.39%
1.29%
GpR-KIMAT1#1
54.57%
36.91%
7.63%
0.88%
PI
FITC
d
H1299
CALU1
Number of colonies
GpR-Ctrl
GpR-KIMAT1#1
GpR-KIMAT1#3
f
H1299
KIMAT1 Fold Change
siCtrl
siKIMAT1#1
siKIMAT1#2
g
H1299
CALU1
siCtrl
siKIMAT1#1
siKIMAT1#2
H1299
CALU1
Number of colonies
siCtrl
siKIMAT1-1
siKIMAT1-2

Supplementary Figure 4: *KIMAT1* is essential for cancer cell survival. a, *KIMAT1* KD efficiency 48h after transfection with a non-targeting GpRs (Ctrl) or three independent GpRs targeting *KIMAT1* in H1299 and CALU6 cells. **= 3.65E-09, 9.09E-08, 5.09E-09 (H1299); 0.000673, 0.000723 and 0.000921 (CALU6) from left to right. **b**, Confocal microscopy images of *KIMAT1* smFISH stained with custom-designed Stellaris probes in H1299 cells upon *KIMAT1* GpRs transfection. Staining performed for DAPI (blue) and *KIMAT1* (red). Scale bar, 75 µm. **c,d**, Colony formation assay (c) and corresponding number of colonies quantification (d) represented as the average number of colonies of three biological replicates ± S.D. upon transfection with *KIMAT1* GpRs. *p* values = 0.0151 and 0.000136 (H1299); 0.014376 and 5.66E-06 (CALU1) from left to right. **e**, *KIMAT1* silencing in CALU1 and H1975 cells induces prominent cell death. **f,** qPCR for *KIMAT1* upon transfection of H1299 cells with two independent siRNAs. **= 4.50E-05 and 2.41E-05 from left to right. **g**, Colony formation assay and corresponding number of colonies quantification in H1299 and CALU1 cells upon *KIMAT1* silencing using two independent *KIMAT1* siRNAs. *p* values= 3.13E-05 and 3.25E-05 (H1299); 0.000147 and 4.52E-05 (CALU1) from left to right. Error bars represent mean ± S.D (n=3), p values were calculated by a two-tailed Student's *t* test.

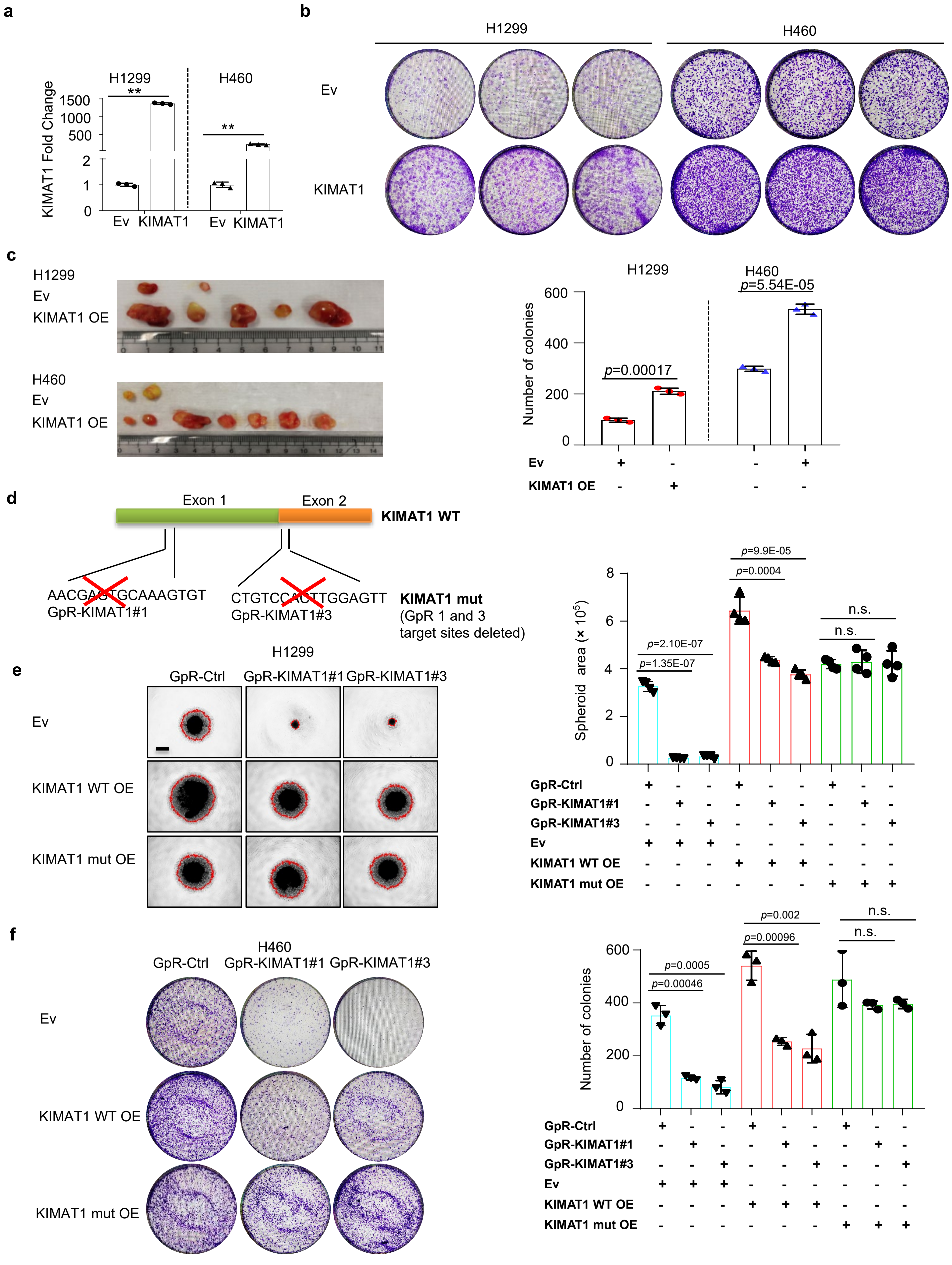
a
H1299
H460
KIMAT1 Fold Change
Ev
KIMAT1
b
H1299
H460
Ev
KIMAT1
c
H1299
Ev
KIMAT1 OE
H460
Ev
KIMAT1 OE
Number of colonies
p=0.00017
p=5.54E-05
d
Exon 1
Exon 2
KIMAT1 WT
AACGAGTGCAAAGTGT
GpR-KIMAT1#1
CTGTCCACTTGGAGTT
GpR-KIMAT1#3
KIMAT1 mut
(GpR 1 and 3
target sites deleted)
e
H1299
GpR-Ctrl
GpR-KIMAT1#1
GpR-KIMAT1#3
Ev
KIMAT1 WT OE
KIMAT1 mut OE
Spheroid area (× 10^5)
p=2.10E-07
p=1.35E-07
p=9.9E-05
p=0.0004
n.s.
f
H460
p=0.0005
p=0.00046
p=0.002
p=0.00096
Number of colonies

Supplementary Figure 5: *KIMAT1* promotes cell proliferation *in vitro* and *in vivo*. **a,** qRT-PCR showing *KIMAT1* induction in cells with stable lentiviral *KIMAT1* OE. **=6.14E-08 (H1299) and 2.59E-05 (H460). **b,** Colony formation assay and corresponding number of colonies quantification (represented as the mean number of colonies of three biological replicates ± S.D.) in H1299 and H460 cell lines with stable *KIMAT1* OE. **= 0.00017 (H1299) and 5.54E-05 (H460). **c**, Photographs of all tumors harvested from NSG mice subcutaneously injected with H1299 and H460 cell lines overexpressing *KIMAT1*. **d,** Schematic representation of the *KIMAT1* sequences deleted to generate GpR resistant (mutant) *KIMAT1***. e-f**, Overexpression of wild-type *KIMAT1* and not of mutant *KIMAT1* (d) halted proliferation (e) and tumorsphere formation (f) in H1299 and H460 cells transfected with *KIMAT1* GpRs. Scale bar, 500 µm. Error bars represent mean ± S.D (a, b and f, n=3; e, n=4), p values were calculated by a two-tailed Student's *t* test.

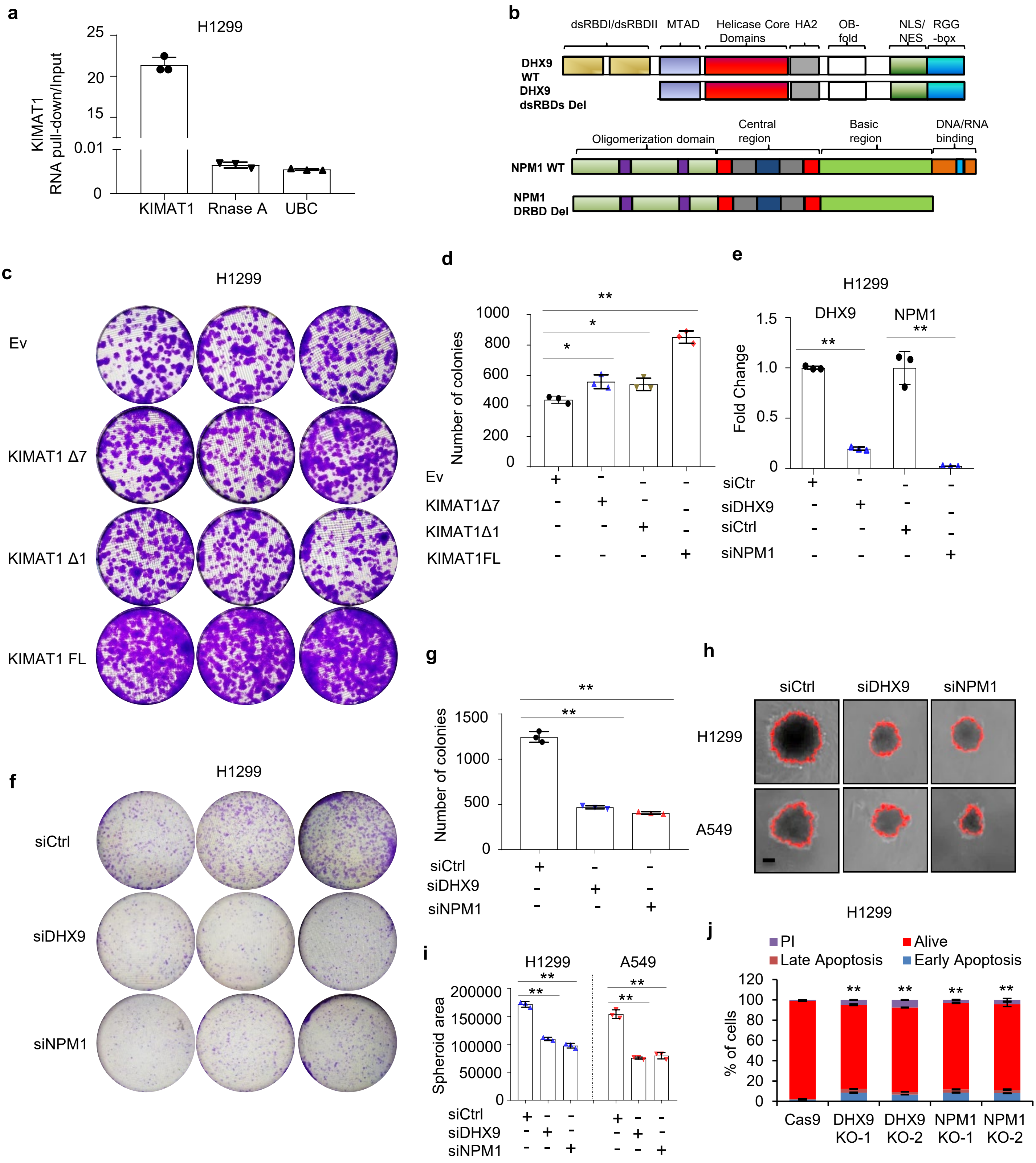
a
H1299
KIMAT1
RNA pull-down/Input
25
20
15
0.01
0
KIMAT1
Rnase A
UBC
b
dsRBDI/dsRBDII
MTAD
Helicase Core Domains
HA2
OB-fold
NLS/NES
RGG-box
DHX9 WT
DHX9 dsRBDs Del
Oligomerization domain
Central region
Basic region
DNA/RNA binding
NPM1 WT
NPM1 DRBD Del
c
H1299
Ev
KIMAT1 Δ7
KIMAT1 Δ1
KIMAT1 FL
d
Number of colonies
1000
800
600
400
200
0
Ev
KIMAT1Δ7
KIMAT1Δ1
KIMAT1FL
e
H1299
DHX9
NPM1
Fold Change
1.5
1.0
0.5
0
siCtr
siDHX9
siCtrl
siNPM1
f
H1299
siCtrl
siDHX9
siNPM1
g
Number of colonies
1500
1000
500
0
siCtrl
siDHX9
siNPM1
h
siCtrl
siDHX9
siNPM1
H1299
A549
i
H1299
A549
Spheroid area
200000
150000
100000
50000
0
siCtrl
siDHX9
siNPM1
j
H1299
PI
Alive
Late Apoptosis
Early Apoptosis
% of cells
120
100
80
60
40
20
0
Cas9
DHX9 KO-1
DHX9 KO-2
NPM1 KO-1
NPM1 KO-2

Supplementary Figure 6: DHX9 and NPM1 mediate *KIMAT1* functional effects. a, Pull-down efficiency of *KIMAT1* using *KIMAT1* biotinylated probes. **b**, Schematic representation of DHX9 and NPM1 functional domains. dsRBD, double-stranded RNA binding domain; MTAD, minimal transactivation domain; HA2, helicase-associated domain 2; OB-fold, oligonucleotide/oligosaccharide-binding fold; NLS, nuclear localization signal; NES, nuclear export signal. **c,d**, Colony formation assay (c) and corresponding number of colonies quantification (d) represented as the mean number of colonies of three biological replicates ± S.D. in H1299 cells upon ectopic expression of either *KIMAT1* full length or deletion constructs. *KIMAT1*Δ7 indicate the fragments without the motif binding to DHX9, *KIMAT1*Δ1 indicates the fragment without the motif binding to NPM1. FL, full length. *p* values= 0.016, 0.021 and 0.0001 from left to right. **e,** qPCR for DHX9 and NPM1 following transfection with a pool of 4 different DHX9 and NPM1 siRNAs. *p* values= 4.67E-07 and 0.0005 from left to right. **f,g**, Clonogenic assay (f) and relative quantification (g) 10-15 days after seeding 5.0 x 10^3 H1299 cells upon transfection with DHX9 or NPM1 siRNA or a non-targeting siRNA, represented as the mean number of colonies of three biological replicates ± S.D. *p* values= 2.69E-05 and 1.87E-05 from left to right. **h,i**, 3D *in vitro* tumorsphere formation assay (h) after transfection with a non-targeting siRNA (siCtrl) or siRNA targeting NPM1 or DHX9 and quantification (i) of the tumorsphere area of three biological replicates ± S.D. *p* valuess= 5.057E-05 and 3.859E-05 (H1299); 7.269E-05 and 0.0001818 (A549) from left to right. Scale bar, 500 μm. **j**, Annexin V assay in two different DHX9 KO and NPM1 KO clones. *p* valuess =1.65E-05, 7.24E-07, 5.52E-06, 2.07E-06 from left to right. Error bars represent mean ± S.D (n=3) in panels with error bars. *p* values were calculated by two-tailed Student's *t* test.

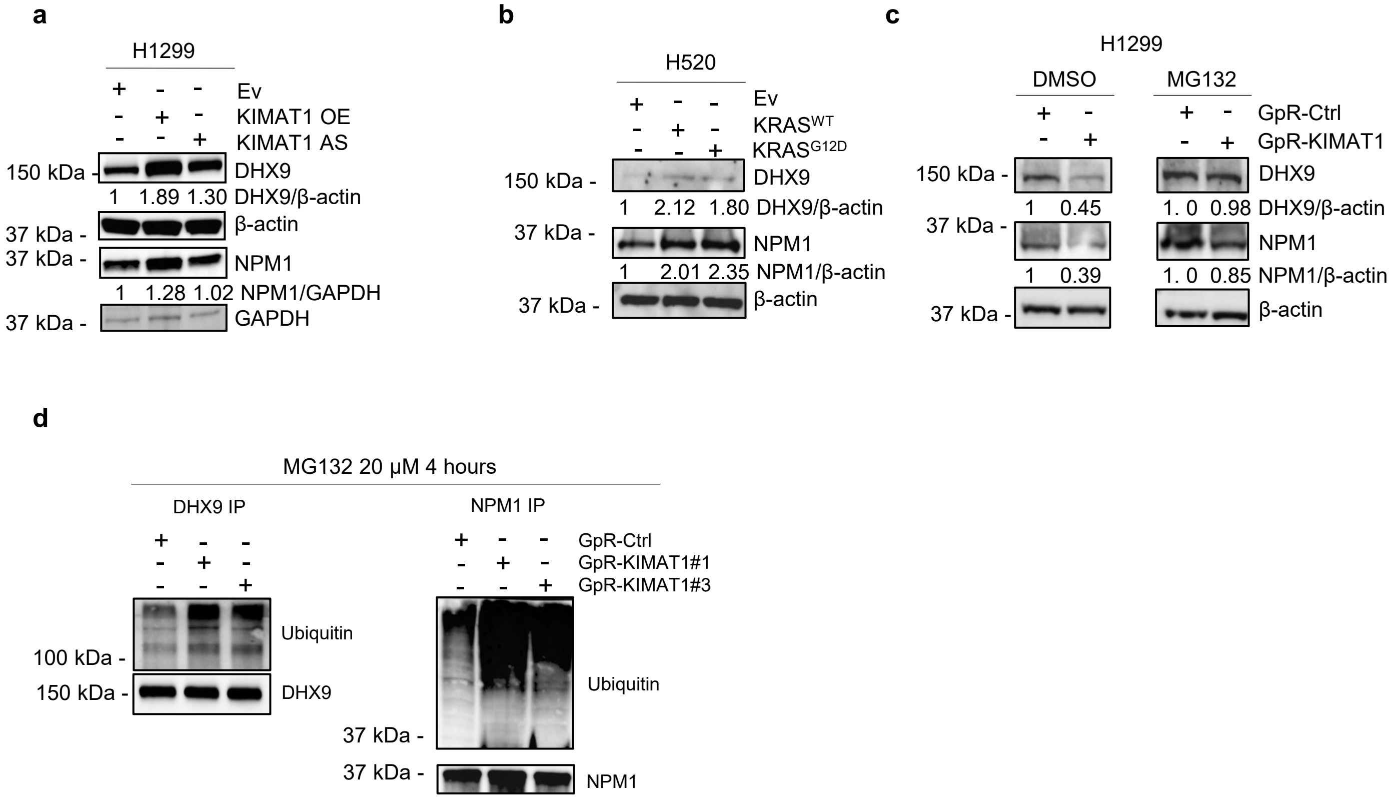

Supplementary Figure 7: *KIMAT1* loss induces ubiquitin-mediated DHX9 and NPM1 degradation. a, DHX9 and NPM1 expression in H1299 cells stably expressing *KIMAT1* or *KIMAT1* antisense. **b**, Ectopic expression of $KRAS^{WT}$ or $KRAS^{G12D}$ induces DHX9 and NPM1 in H520 squamous cell carcinoma cells. **c,** Downregulation of DHX9 and NPM1 caused by *KIMAT1* silencing is rescued by MG132. **d,** KIMAT1 silencing resulted in increased polyubiquitination of DHX9 and NPM1 in H1299 cells. a-d, Representative images of 2 biological replicates.

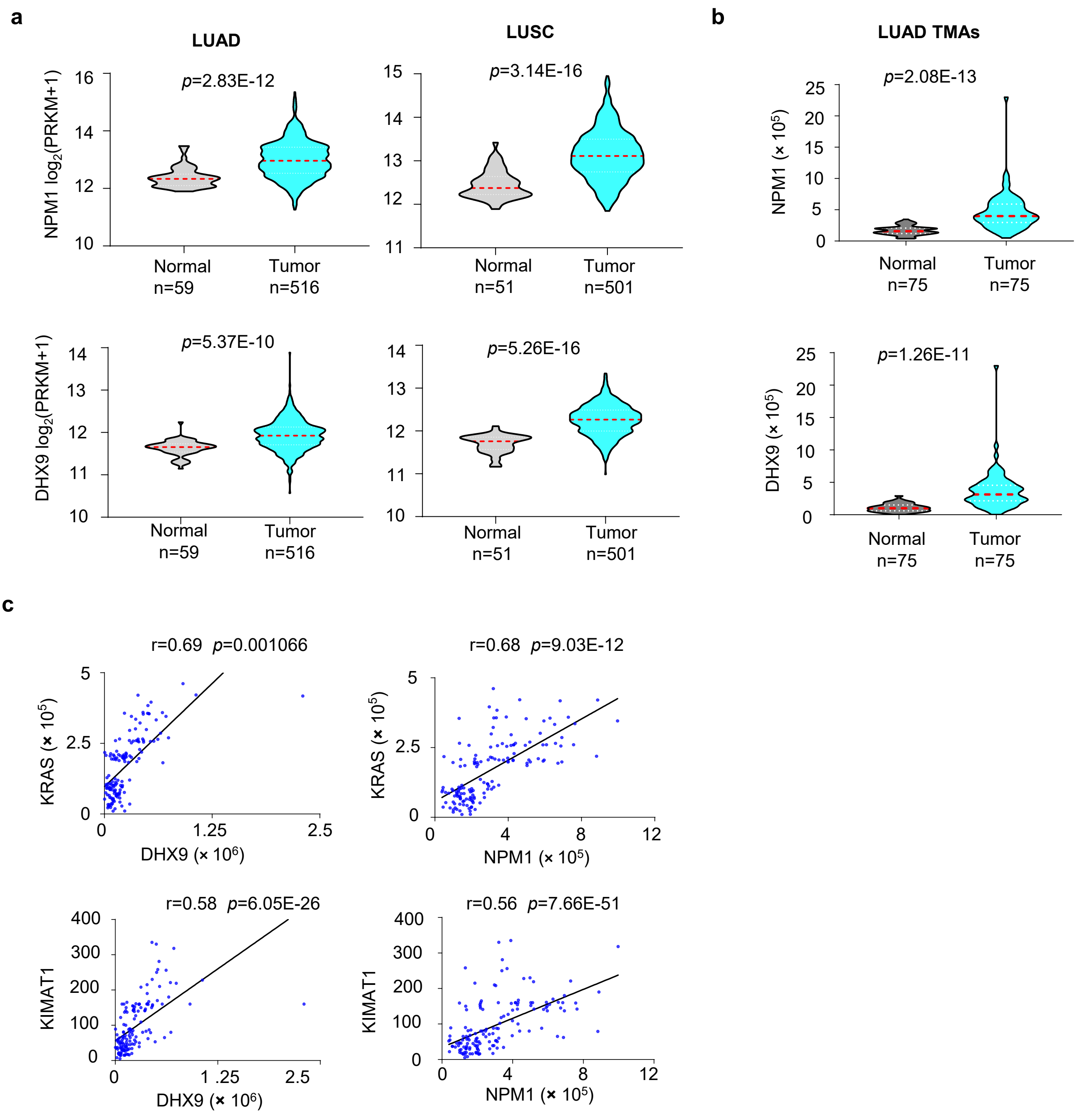

Supplementary Figure 8: DHX9 and NPM1 are enriched in tumors versus normal lung and positively correlate with KRAS and *KIMAT1*. a, DHX9 and NPM1 are enriched in LUAD and LUSC lesions compared to normal lung. **b,c,** DHX9 and NPM1 are enriched in FFPE lung adenocarcinoma lesions compared to normal lung and positively correlate with KRAS and *KIMAT1* by Pearson's rank correlation coefficient (*r*). *p* values were calculated by two-tailed Student's *t* test.

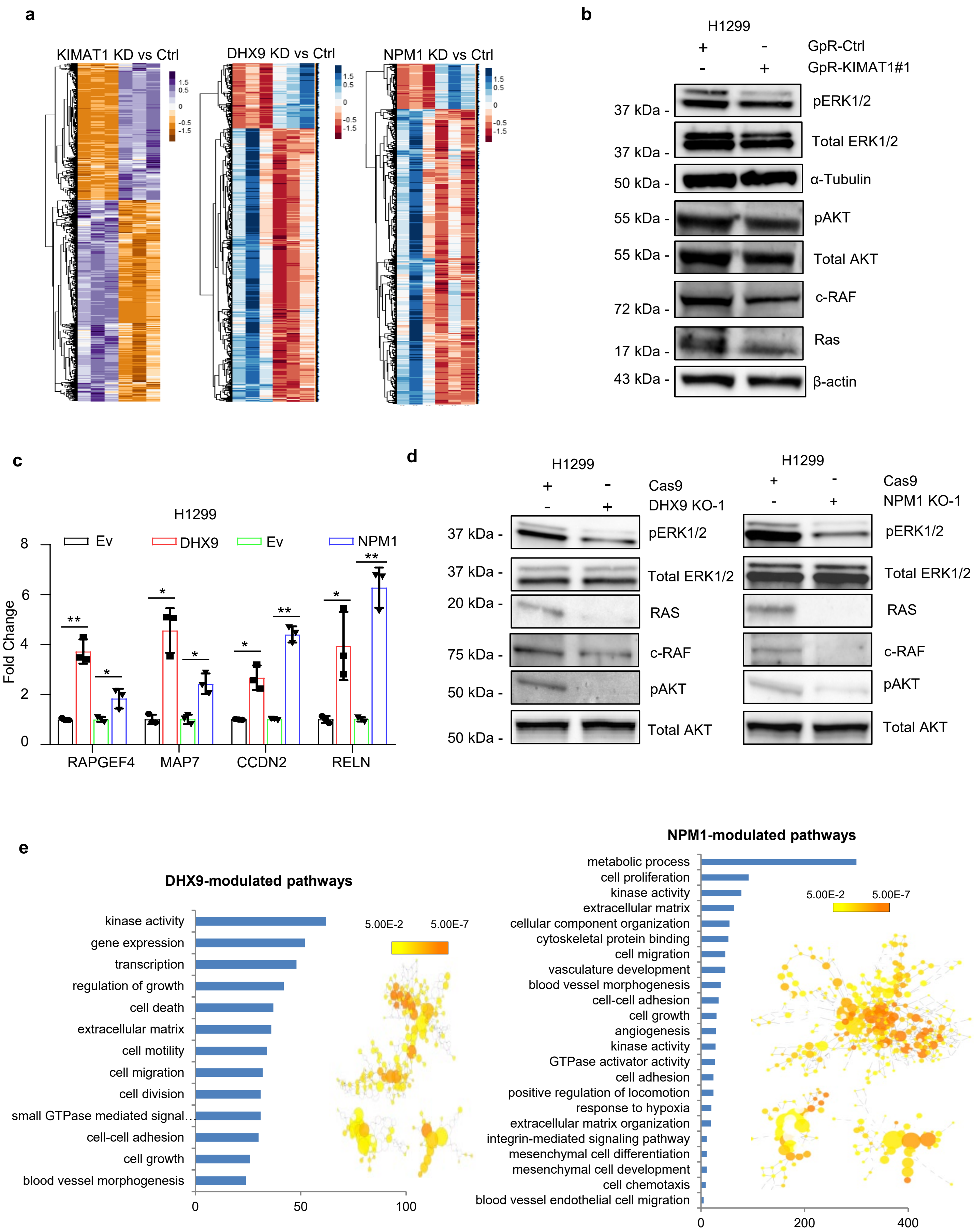

a
KIMAT1 KD vs Ctrl
DHX9 KD vs Ctrl
NPM1 KD vs Ctrl
b
H1299
GpR-Ctrl
GpR-KIMAT1#1
pERK1/2
Total ERK1/2
α-Tubulin
pAKT
Total AKT
c-RAF
Ras
β-actin
37 kDa -
37 kDa -
50 kDa -
55 kDa -
55 kDa -
72 kDa -
17 kDa -
43 kDa -
c
H1299
Ev
DHX9
Ev
NPM1
Fold Change
RAPGEF4
MAP7
CCDN2
RELN
d
H1299
Cas9
DHX9 KO-1
H1299
Cas9
NPM1 KO-1
pERK1/2
Total ERK1/2
RAS
c-RAF
pAKT
Total AKT
37 kDa -
37 kDa -
20 kDa -
75 kDa -
50 kDa -
50 kDa -
e
DHX9-modulated pathways
kinase activity
gene expression
transcription
regulation of growth
cell death
extracellular matrix
cell motility
cell migration
cell division
small GTPase mediated signal…
cell-cell adhesion
cell growth
blood vessel morphogenesis
5.00E-2
5.00E-7
NPM1-modulated pathways
metabolic process
cell proliferation
kinase activity
extracellular matrix
cellular component organization
cytoskeletal protein binding
cell migration
vasculature development
blood vessel morphogenesis
cell-cell adhesion
cell growth
angiogenesis
kinase activity
GTPase activator activity
cell adhesion
positive regulation of locomotion
response to hypoxia
extracellular matrix organization
integrin-mediated signaling pathway
mesenchymal cell differentiation
mesenchymal cell development
cell chemotaxis
blood vessel endothelial cell migration
5.00E-2
5.00E-7

Supplementary Figure 9: Genes and pathways modulated by *KIMAT1*, DHX9 and NPM1. a, Heatmaps showing dysregulated genes upon *KIMAT1,* DHX9 or NPM1 silencing using GpRs (*KIMAT1*) or a pool of 4 different siRNAs (DHX9 and NPM1). **b**, Immunoblotting with the indicated antibodies upon *KIMAT1* silencing. Representative images of two biological replicates. **c,** qPCR analysis of KRAS target genes upon DHX9 or NPM1 KD in H1299 cells. Error bars represent mean ± S.D (n=3), p values were calculated by a two-tailed Student's *t* test. *p* values= 0.0006, 0.024, 0.003, 0.006, 0.004, 5.35E-05, 0.021 and 0.0004 from left to right. **d**, Immunoblotting with the indicated antibodies in DHX9 KO or NPM1 KO cells. Representative images of two biological replicates. **e**, Gene Ontology (GO) enrichment analysis was performed for the significant differentially expressed genes (DHX9, n=5277; NPM1, n=6279) after DHX9 or NPM1 silencing using BINGO. GO themes involved in biological processes (GOBP), molecular functions (GOMF), and cellular components (GOCC) are shown. GO themes were sorted based on significant *p*-values. The color intensity of nodes refers to the corrected *p* value of ontology. The size of nodes refers to the numbers of genes that are involved in the ontology. $p < 0.05$ was considered statistically significant by a two-tailed Student's *t* test.

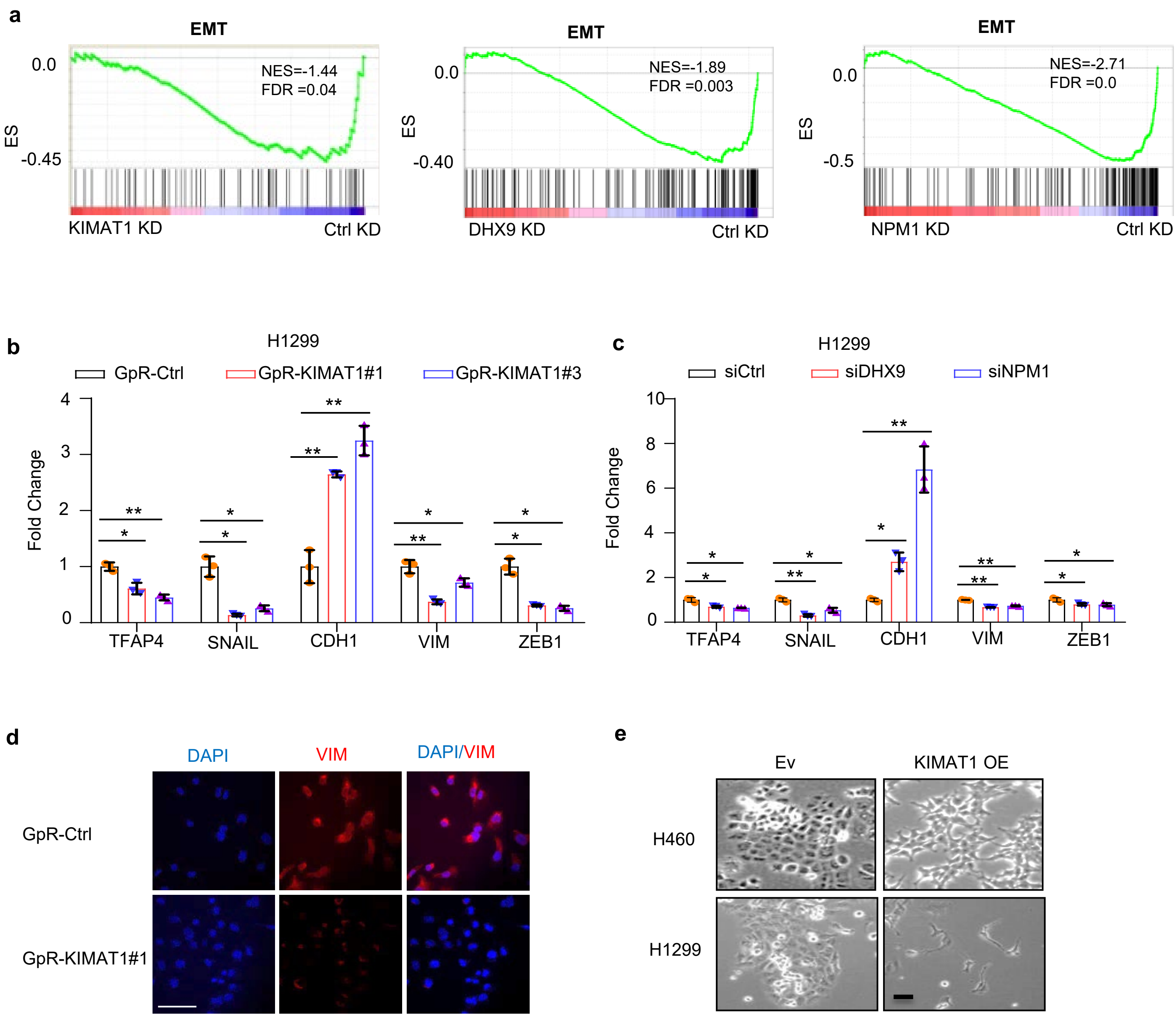

a
EMT
NES=-1.44
FDR =0.04
ES
0.0
-0.45
KIMAT1 KD
Ctrl KD
EMT
NES=-1.89
FDR =0.003
0.0
-0.40
DHX9 KD
Ctrl KD
EMT
NES=-2.71
FDR =0.0
0.0
-0.5
NPM1 KD
Ctrl KD
b
H1299
GpR-Ctrl
GpR-KIMAT1#1
GpR-KIMAT1#3
Fold Change
TFAP4
SNAIL
CDH1
VIM
ZEB1
c
H1299
siCtrl
siDHX9
siNPM1
Fold Change
TFAP4
SNAIL
CDH1
VIM
ZEB1
d
DAPI
VIM
DAPI/VIM
GpR-Ctrl
GpR-KIMAT1#1
e
Ev
KIMAT1 OE
H460
H1299

Supplementary Figure 10: *KIMAT1*, DHX9 and NPM1 regulate EMT. a, GSEA analysis showing enrichment of the EMT signature in *KIMAT1*-, DHX9- and NPM1-regulated genes. **b,c**, qPCR for several EMT markers upon *KIMAT1*, DHX9 or NPM1 silencing. Error bars represent mean ± S.D (n=3), p values were calculated by a two-tailed Student's *t* test. p values= 0.006, 0.0005, 0.001, 0.002, 0.0007, 0.0006, 0.00097, 0.0242, 0.0011 and 0.001 (b); 0.009, 0.004, 0.0003, 0.004, 0.002, 0.0006, 1.49E-05, 8.25E-05, 0.037 and 0.038 (c) from left to right. **d**, Vimentin expression by immunofluorescence (red) in cells transfected with GpR-Ctrl or with GpR-*KIMAT1*. 40 x magnification. Scale bar, 75 μm. Images are representative of two biological replicates. **e**, Representative photomicrographs depicting a morphological change in H460 and H1299 cells stably overexpressing *KIMAT1*. Scale bar, 500 μm.

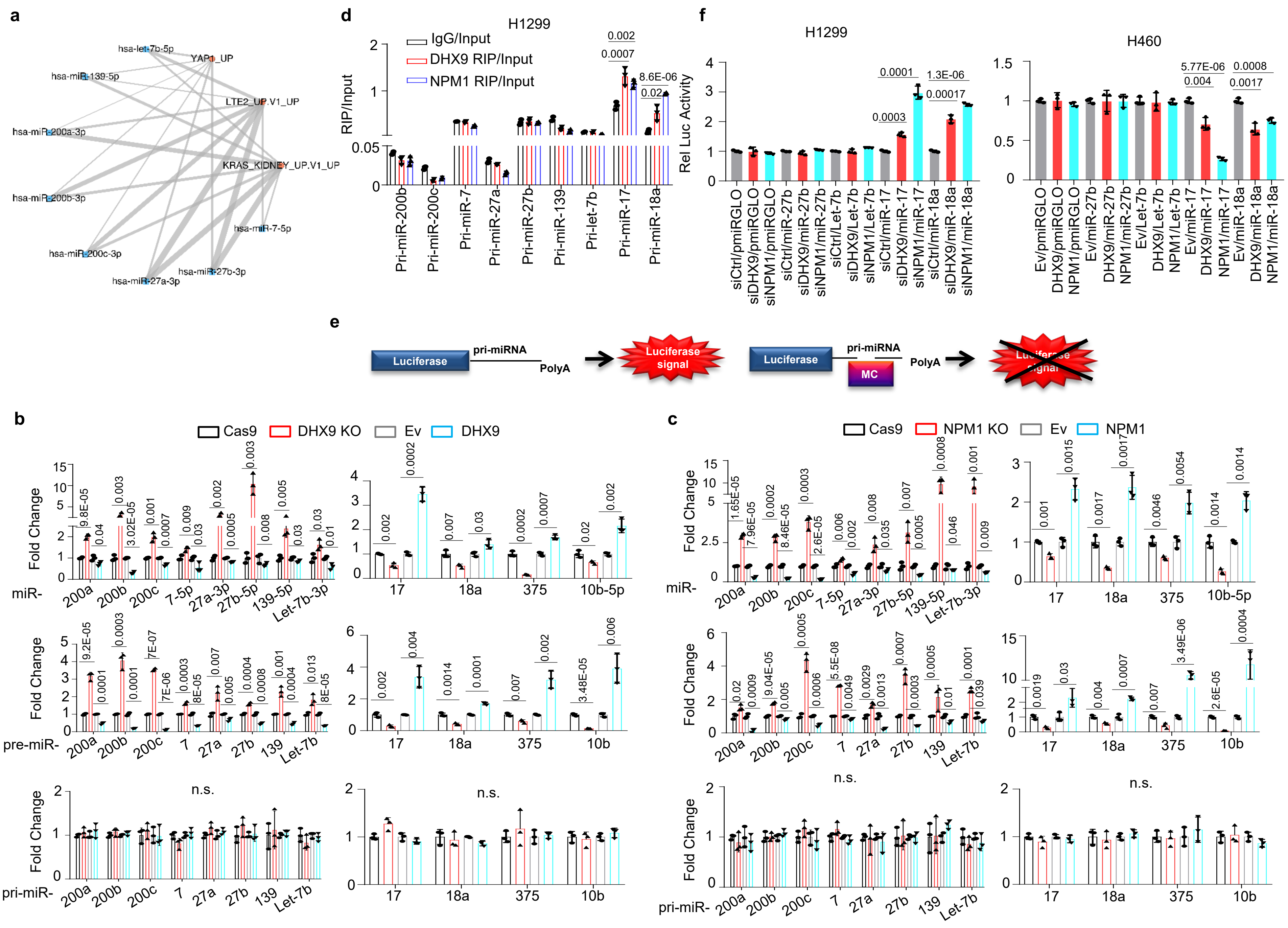

a
b
c
d
e
f
H1299
H460
IgG/Input
DHX9 RIP/Input
NPM1 RIP/Input
RIP/Input
Rel Luc Activity
Luciferase
pri-miRNA
PolyA
Luciferase signal
MC
Cas9
DHX9 KO
Ev
DHX9
NPM1 KO
NPM1
Fold Change
miR-
pre-miR-
pri-miR-
n.s.

Supplementary Fig. 11: DHX9 and NPM1 control miRNA processing. a, Directed Network Diagram of Enriched Pathways regulated by *KIMAT1* within Predicted miRNA Targets derived from KIMAT1 KD (DEGs with FDR<0.05 which are also predicted to be miRNA targets by Targetscan [release 7.2 – default predictions]). The statistical method to obtain the DEGs has been described in the legend of fig. 6a. The diagram displays miRNAs (blue squares) and pathways enriched (orange circles) (FDR<0.05; calculated using GSEA with DEGs from KIMAT1 KD) and the connecting edges (gray). Edge-widths represent the number of DEGs contributing to the connection within each gene set. The network graph was produced using the igraph package (version 1.2.4.2). **b**, Expression levels of primary, precursors and mature forms of the indicated miRNAs were analysed by qPCR in DHX9 KO cells or in cells transfected with a DHX9 expression vector. Primary and precursor miRNAs were normalized to *β-actin*, and mature miRNAs were normalized to *RNU48.* **c,** Expression levels of primary, precursors and mature forms of the indicated miRNAs were analysed by qPCR after NPM1 silencing or overexpression in H1299 cells. Primary and precursor miRNAs were normalized to *β-actin* and mature miRNAs were normalized to *RNU48.* **d,** CLIP showing enrichment of TP and not TS miRNAs upon DHX9 or NPM1 pulldown. **e,** Schematic diagram of *in vivo* cellular monitoring assay of DHX9 and NPM1 function. MC=Microprocessor complex. **f,** H1299 and H460 cell lines were transfected with a luciferase vector carrying a segment of *pri-miR-27b*, *pri-let-7b*, *pri-miR-17* or *pri-miR-18a* between the luciferase gene and the polyadenylation signal. DHX9 or NPM1 silencing caused an increase of the luciferase activity in cells transfected with *pri-miR-17* or *pri-miR18a* and not in cells transfected with *pri-miR-27b* or *pri-let7b*. DHX9 or NPM1 OE sorted the opposite effect. Error bars represent mean ± S.D (n=3), *p* values were calculated by a two-tailed Student's *t* test.

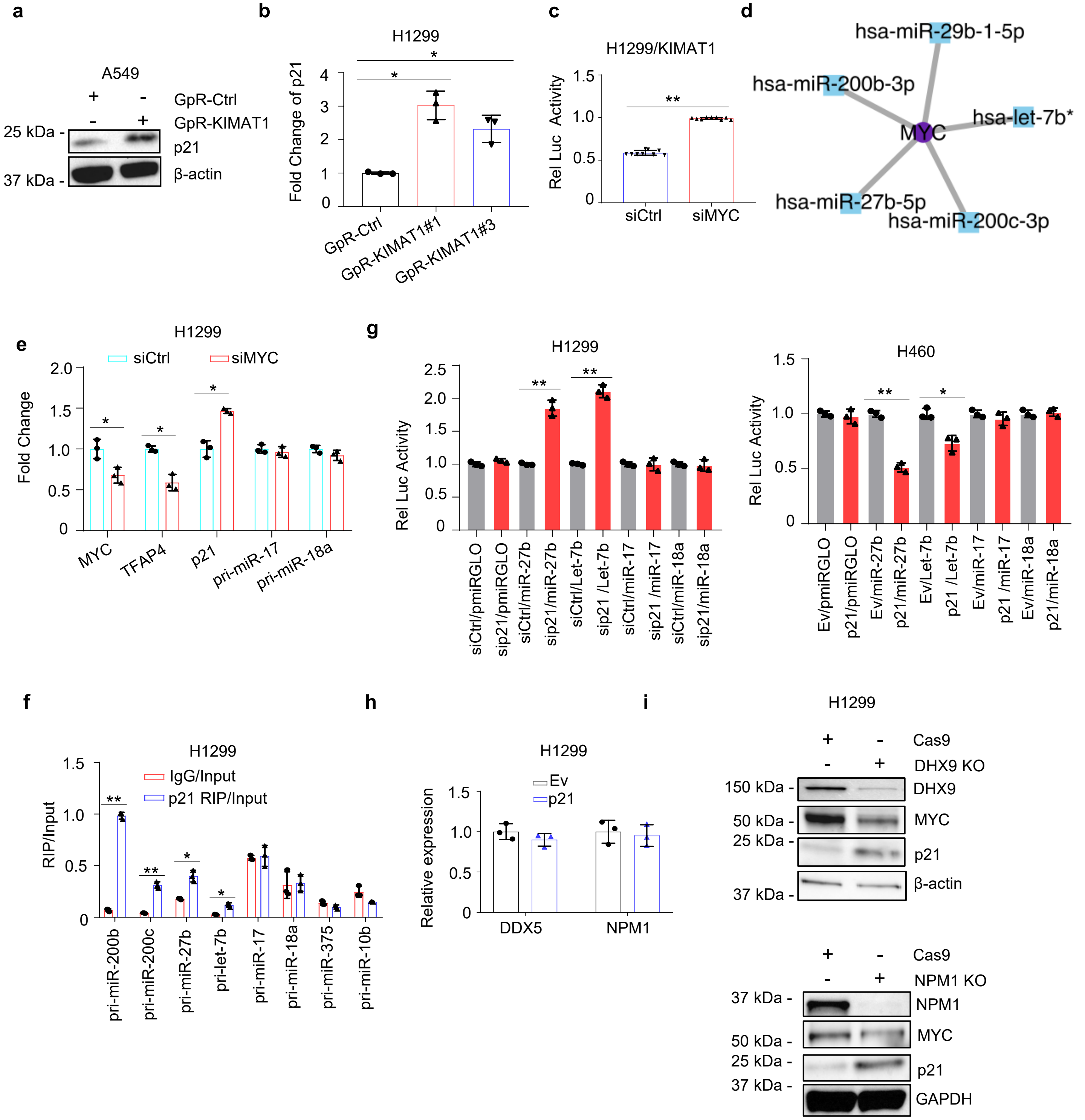
a
A549
GpR-Ctrl
GpR-KIMAT1
25 kDa -
p21
37 kDa -
β-actin
b
H1299
Fold Change of p21
GpR-Ctrl
GpR-KIMAT1#1
GpR-KIMAT1#3
c
H1299/KIMAT1
Rel Luc Activity
siCtrl
siMYC
d
hsa-miR-29b-1-5p
hsa-miR-200b-3p
hsa-let-7b*
MYC
hsa-miR-27b-5p
hsa-miR-200c-3p
e
H1299
siCtrl
siMYC
Fold Change
MYC
TFAP4
p21
pri-miR-17
pri-miR-18a
g
H1299
Rel Luc Activity
siCtrl/pmiRGLO
sip21/pmiRGLO
siCtrl/miR-27b
sip21/miR-27b
siCtrl/Let-7b
sip21 /Let-7b
siCtrl/miR-17
sip21 /miR-17
siCtrl/miR-18a
sip21/miR-18a
H460
Rel Luc Activity
Ev/pmiRGLO
p21/pmiRGLO
Ev/miR-27b
p21/miR-27b
Ev/Let-7b
p21 /Let-7b
Ev/miR-17
p21 /miR-17
Ev/miR-18a
p21/miR-18a
f
H1299
IgG/Input
p21 RIP/Input
RIP/Input
pri-miR-200b
pri-miR-200c
pri-miR-27b
pri-let-7b
pri-miR-17
pri-miR-18a
pri-miR-375
pri-miR-10b
h
H1299
Ev
p21
Relative expression
DDX5
NPM1
i
H1299
Cas9
DHX9 KO
150 kDa -
DHX9
50 kDa -
MYC
25 kDa -
p21
37 kDa -
β-actin
Cas9
NPM1 KO
37 kDa -
NPM1
50 kDa -
MYC
25 kDa -
p21
37 kDa -
GAPDH

Supplementary Figure 12: p21 binds and promotes the processing of tumor suppressive miRNAs. **a**,**b,** p21 protein and mRNA induction upon *KIMAT1* KD. *p* values =0.0012 (KIMAT1#1 vs Ctrl) and 0.005 (KIMAT1#3 vs Ctrl). n=2**. c**, Luciferase reporter assay of p21 promoter activity upon MYC silencing in H1299/*KIMAT1* stable cell lines. n=9, **= 1.68E-17. **d**, Network depicting *KIMAT1* repressed miRNAs targeting MYC using Targetscan and Diana-TarBase algorithms. **e**, MYC silencing does not affect *pri-miR-17* and *pri-miR-18a*. TFAP4 and p21 were used as positive controls. *p* values =0.022, 0.003 and 0.002 from left to right. **f**, p21 binds to TS and not to TP miRNAs by CLIP. *p* values= 1.63E-06, 0.0001, 0.003 and 0.002 from left to right. **g**, *In vivo* cellular monitoring assay in H1299 and H460 cell lines transfected with a luciferase vector carrying a segment of *pri-miR-27b*, *pri-let-7b*, *pri-miR-17* or *pri-miR-18a* between the luciferase gene and polyadenylation signal. *p* values= 0.00027 and 4.11E-05 (Left); 8.4E-05 and 0.006 (Right) from left to right. **h,** p21 enforced expression does not affect *DDX5* and *NPM1* mRNA**.** *p* values=0.24 and 0.69**. i,** Immunobloting with the indicated antibodies in DHX9 and NPM1 KO cells. n=2 Error bars represent mean ± S.D (n=3), *p* values were calculated by a two-tailed Student's *t* test.

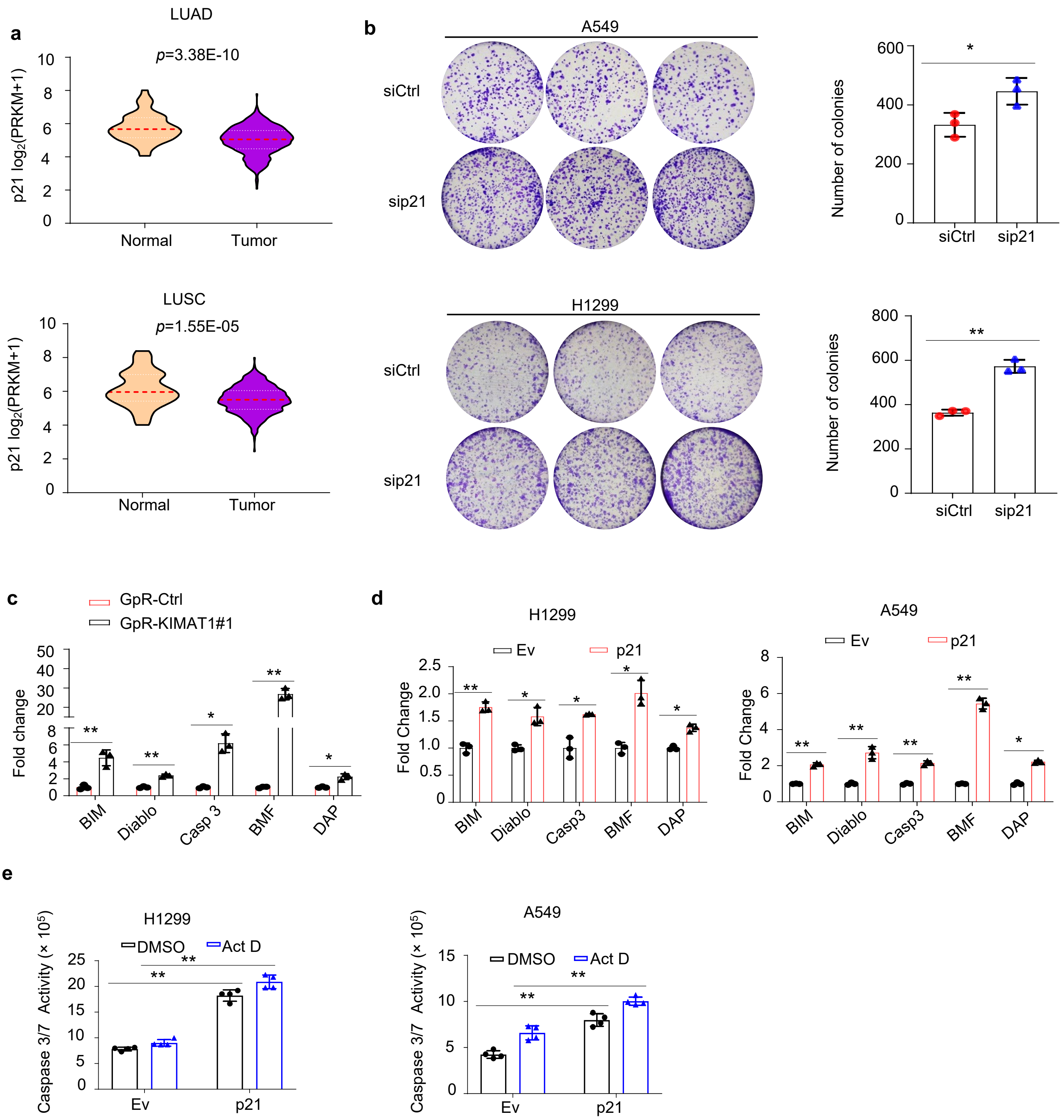
a
LUAD
p=3.38E-10
p21 log2(PRKM+1)
Normal
Tumor
LUSC
p=1.55E-05
b
A549
siCtrl
sip21
Number of colonies
H1299
c
GpR-Ctrl
GpR-KIMAT1#1
Fold change
BIM
Diablo
Casp 3
BMF
DAP
d
H1299
Ev
p21
Fold Change
Casp3
A549
e
Caspase 3/7 Activity (× 10^5)
DMSO
Act D

Supplementary Figure 13: p21 is downregulated in LUAD and LUSC and its overexpression induces cell death. a, p21 is downregulated in the LUAD and LUSC datasets (TCGA) compared to normal lung samples. *p* value was calculated by a two-tailed Student's *t* test. **b,** Colony formation assay and corresponding quantification in A549 (*=0.03) and H1299 (**=0.0004) cells upon p21 silencing. **c,d,** qRT-PCR of pro-apoptotic genes upon *KIMAT1* silencing (c) or p21 OE (d) in p53 null or p53 wild-type cells. BMF, Bcl2 modifying factor; DAP, death-associated protein 1. *p* values= 0.004, 0.0002, 0.001, 9.97E-05 and 0.004 (C); 0.0006, 0.005, 0.005, 0.002 and 0.001 (d, Left); 4.51E-05, 0.00097, 0.0001, 1.41E-05 and 4.51E-05 (d, Right) from left to right. **e,** Treatment of H1299 and A549 cells with actinomycin D (Act D) does not suppress p21-mediated apoptosis. *p* values = 1.94E-06 and 3.47E-06 (Left); 8.15E-05 and 0.00024 (Right) from left to right. b-e, Error bars represent mean ± S.D (n=3), p values were calculated by a two-tailed Student's *t* test.

a

PDX	IC11LC13
**Biopsy origin**	Lung squamous cell carcinoma (primary tumor)
**Treatment before biopsy**	no chemotherapy
**KRAS status**	WT amplification (six copies)
**NRAS**	WT
**P53 status**	E162fs mutation (homozygous)

b

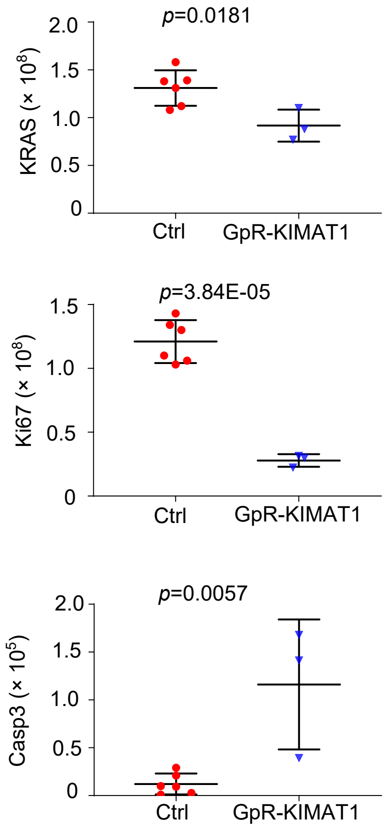

c

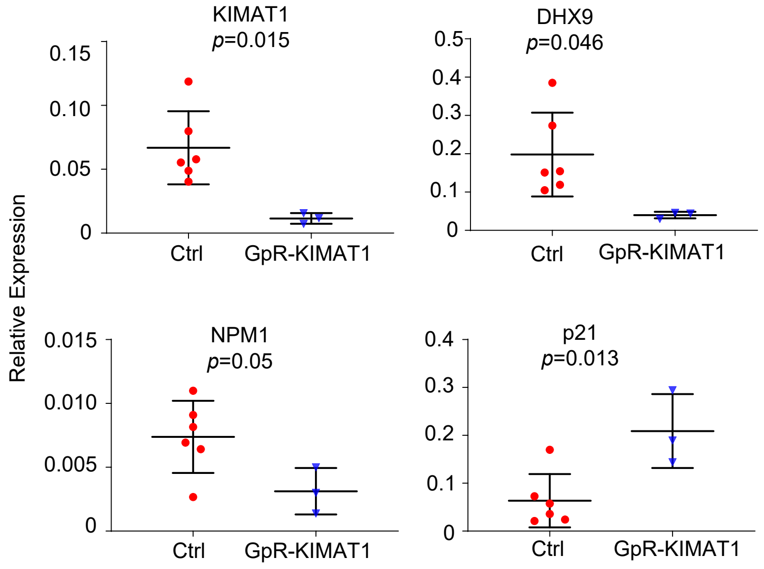

Supplementary Figure 14: *KIMAT1* KD reduces tumor growth in a PDX mouse model. a, Table indicates the mutational status of the PDX used in the study. **b**, Quantification of KRAS, Ki67 and cleaved caspase 3 in mice treated with *KIMAT1* GpRs compared to control mice by IHC analysis. **c**, qPCR for *KIMAT1*, DHX9, NPM1 and p21 in tumors derived from the PDX mouse model reported in a. Ctrl n=6 *KIMAT1* KD n=3. Error bars represent mean ± S.D, p values were calculated by two-tailed Student's*t* test.

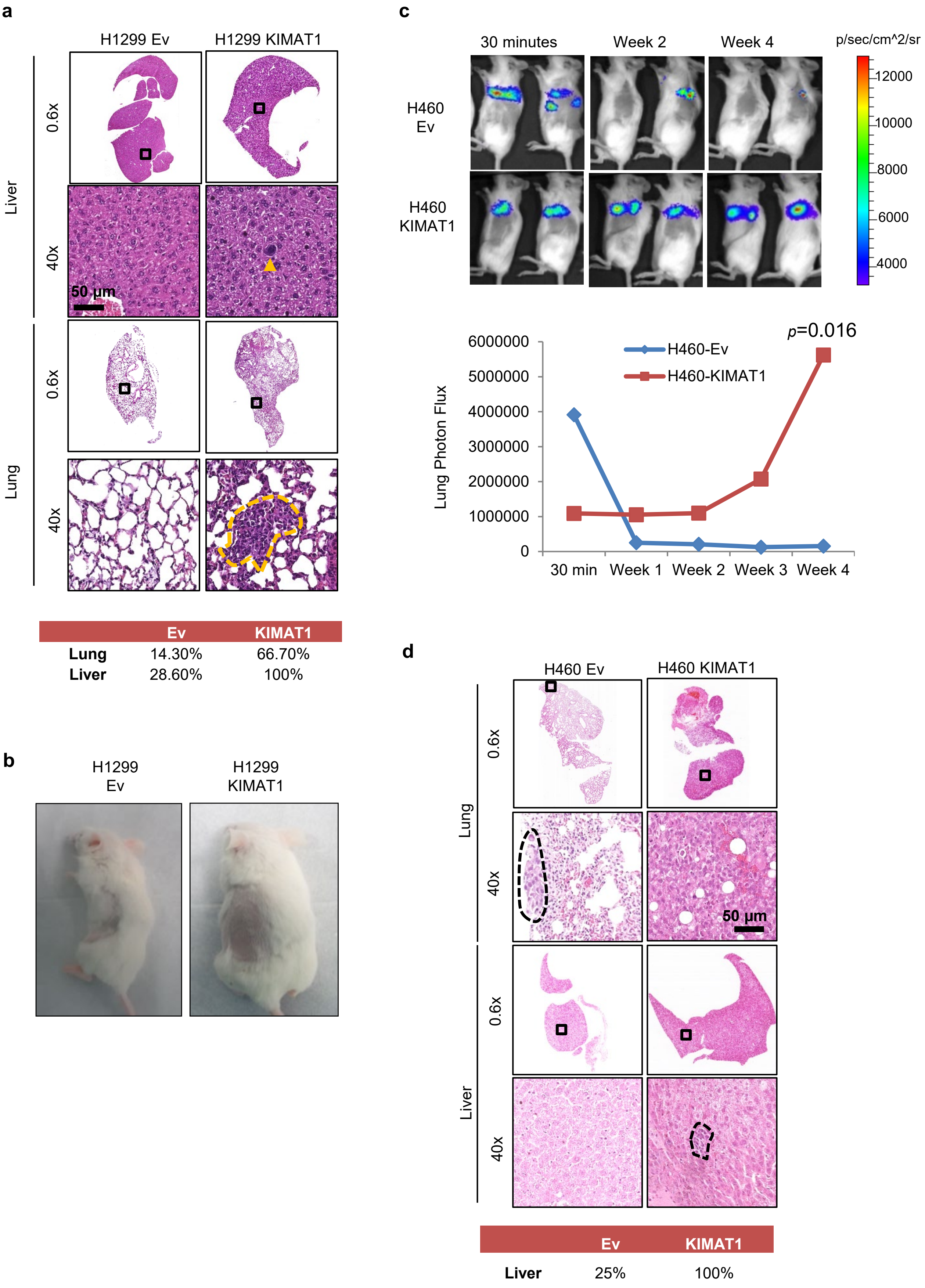

	Ev	KIMAT1
**Lung**	14.30%	66.70%
**Liver**	28.60%	100%

	Ev	KIMAT1
**Liver**	25%	100%

Supplementary Figure 15: *KIMAT1* overexpression promotes distant metastases *in vivo*. a, Representative lung and liver sections from NSG mice injected i.v. with cells stably overexpressing *KIMAT1* or an empty vector. A single isolated tumor cell (arrow's head) and a small neoplastic lesion are shown in the liver and lungs of a H1299/KIMAT1 mouse, respectively. Scale bar, 50 μm. The table reports the percentage of mice from the two groups with neoplastic lesions in the lungs and liver metastases. **b**, Representative images of mice orthotopically implanted with H1299/*luc+*/*KIMAT1* stable cells with malignant ascites before sacrifice. a,b, n=7. **c,** Bioluminescent signals (top) and quantification (bottom) of lung tumors derived from mice injected with H460*luc2+/KIMAT1* cells relative to control mice. Error bars represent mean ± S.D (n=8). Two-tailed Student's *t* test was used to measure statistical significance. **d**, Representative H&E images of lungs and liver for the mice in c. Mice injected with H460/luc+/KIMAT1 stable cell lines present a large neoplastic area in the lungs (upper panels) and a small metastatic locus (dashed lines) in the liver. A small lesion (dashed lines) is observed in the lungs of control mice, no metastases or micrometastases are evident in the liver. Scale bar, 50 μm The table underneath the panels report the percentage of mice from the two groups with liver micrometastases. n=8.

a

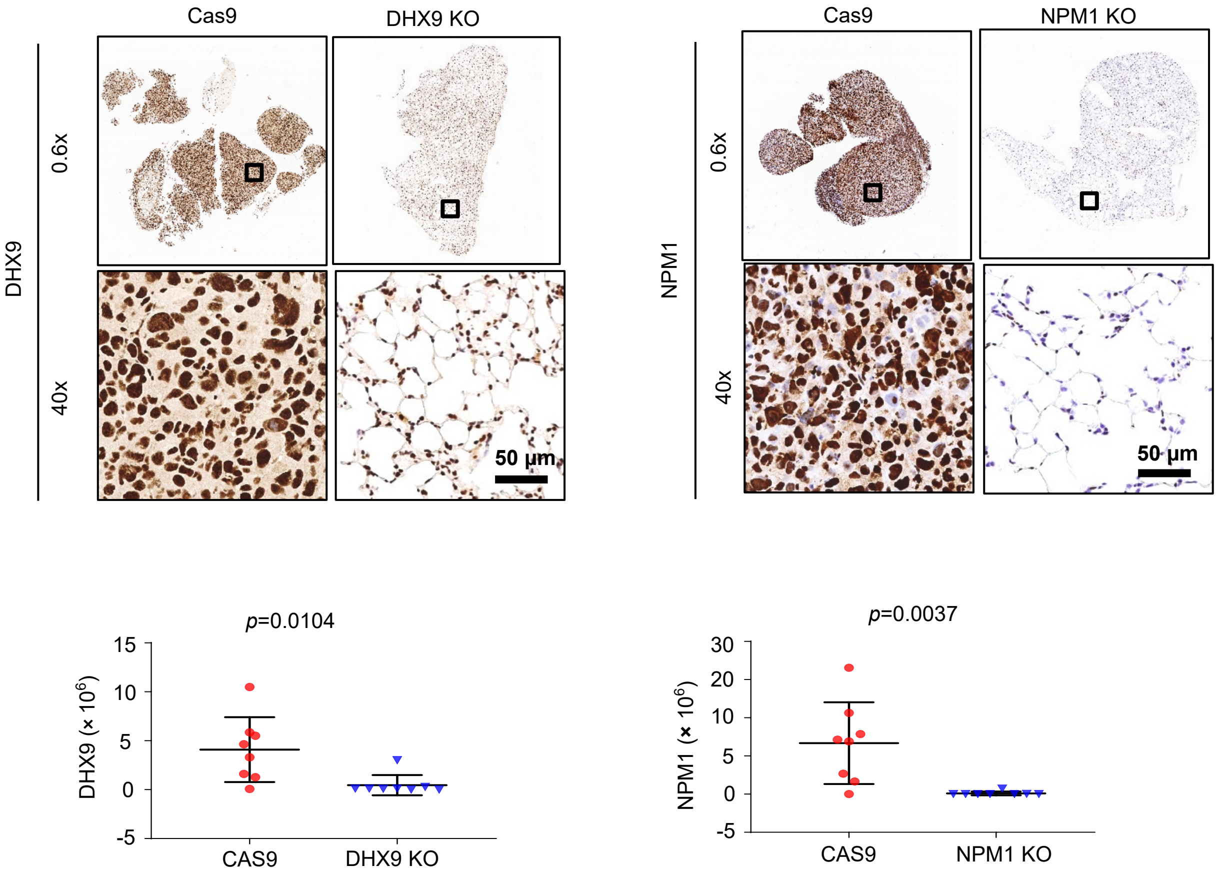

b

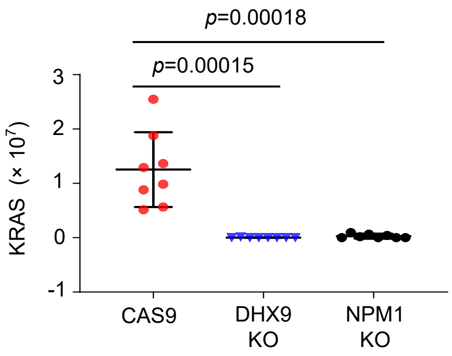

c

	Cas9	DHX9 KO	NPM1 KO
**Lung**	87.5%	37.5%	0%
**Liver**	50%	12.5%	25%

Supplementary Figure 16: DHX9 KO and NPM1 KO abrogate distant metastases in vivo.a, Representative IHC (top) and corresponding quantification (bottom) of DHX9 and NPM1 expression in lungs of mice orthotopically injected with DHX9 KO, NPM1 KO or control cells (Cas9). **b**, Relative expression of KRAS in lungs of mice orthotopically injected with DHX9 KO or NPM1 KO cells. **c,** Table reporting the percentage of micrometastatic loci in mice orthotopically injected with DHX9 KO, NPM1 KO or control cells (Cas9). Error bars represent mean ± S.D (n=8), *p* values were calculated by a two-tailed Student's *t* test.

a

Mice	Distended abdomens/ Ascites
Ev+Cas9	2/8
KIMAT1 OE + Cas9	6/8
KIMAT1 OE + DHX9 KO	3/8
KIMAT1 OE + NPM1 KO	2/8

c

	lung	liver
Ev + Cas9	44.88%	6.68%
KIMAT1 OE + Cas9	72.63%	56.88%
KIMAT1 OE + DHX9 KO	42.63%	36%
KIMAT1 OE + NPM1 KO	41.25%	20.75%

b

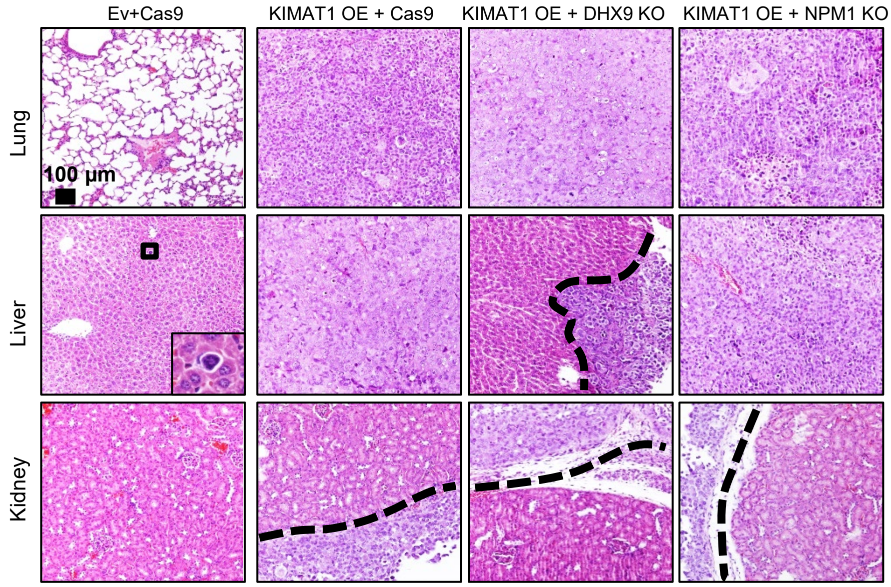

d

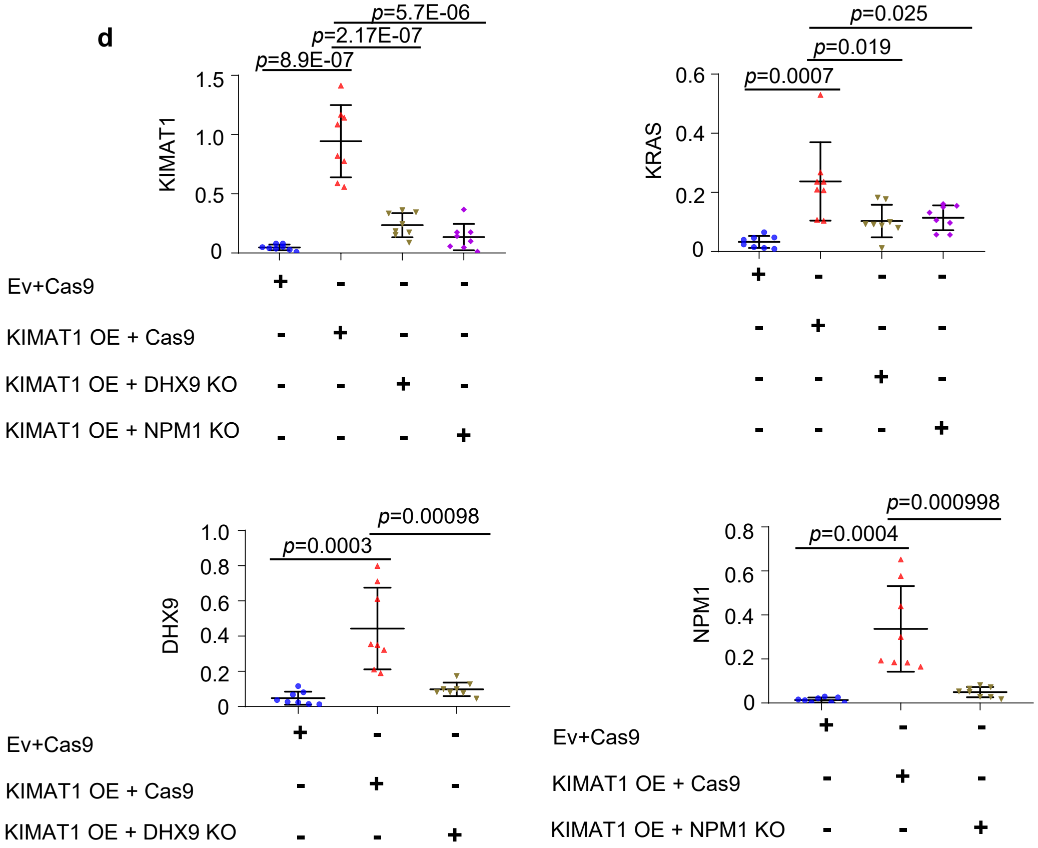

Supplementary Figure 17: DHX9 KO or NPM1 KO halted *KIMAT1*-mediated distant metastases. **a**, Clinical symptoms in mice injected orthotopically with the indicated cell lines. **b**, Representative H&E images of lungs, liver and kidney for the four groups of mice injected with the indicated cells. Mice injected with *KIMAT1* OE+Cas9 stable cells presented a large neoplastic area in the lungs and metastatic involvement in the liver and in the perirenal fat. Mice injected with Ev+Cas9 stable cell lines showed a lower metastatic rate. Note the single tumor cell highlighted (inset) in the liver of a mouse injected with Ev+Cas9 cells (Original magnification 10x; scale bar, 50 μm). **c**, The table reports the rate of mice showing neoplastic area in the lungs and metastasis in the liver from the four groups. **d**, qPCR analysis for the indicated genes in tumor derived from a. Error bars represent mean ± S.D (n=8), $^{**}p$ value < 0.001, $^{*}p$ value < 0.05 by a two-tailed Student's *t* test.

Supplementary Table 1. siRNAs and GapmeRs

**siRNA**	**Source**	**Catalog number**	**Assay ID**
siDHX9	Thermofisher Scientific	4390824	s4020
siKRAS	Thermofisher Scientific	4390824	s7938
siRNA Negative Control	Thermofisher Scientific	4390843	N/A
SMARTpool: ON-TARGETplus NPM1 siRNA	Dharmacon	L-015737-00-0005	N/A
SMARTpool: ON-TARGETplus CDKN1A siRNA	Dharmacon	L-003471-00-0005	N/A
SMARTpool: ON-TARGETplus MYC siRNA	Dharmacon	L-003282-02-0005	N/A
SMARTpool: ON-TARGETplus EGFR siRNA	Dharmacon	L-003114-00-0005	N/A
ON-TARGETplus Non-targeting Pool	Dharmacon	D-001810-10-05	N/A

**lncRNA**	**Source**	**Sequence**
Negative Control	QIAGEN	AACACGTCTATACGC
GapmeR KIMAT1#1	QIAGEN	AACGAGTGCAAAGTGT
GapmeR KIMAT1#2	QIAGEN	TCTGTGGTGTGCTCTT
GapmeR KIMAT1#3	QIAGEN	CTGTCCACTTGGAGTT

Supplementary Table 2. lncRNA FISH and RAP-MS probes

KIMAT1 FISH probes

**Probe ID**	**Sequence**	**Modification**
KIMAT1 FISH_1	CTGATGCGTTCCTCTCAG	CAL Fluor Red 590
KIMAT1 FISH_2	CAGCCGCTTGTTTTCTTC	CAL Fluor Red 590
KIMAT1 FISH_3	CTTGACGTCCTCTCCACG	CAL Fluor Red 590
KIMAT1 FISH_4	GTCTGTGGTGTGCTCTTC	CAL Fluor Red 590
KIMAT1 FISH_5	AAACTCCGCCTGGTCCTG	CAL Fluor Red 590
KIMAT1 FISH_6	CTTAGTGGCCTAGCTCTC	CAL Fluor Red 590
KIMAT1 FISH_7	GGTTTATCTCCTGGAGTT	CAL Fluor Red 590
KIMAT1 FISH_8	CGACGGAGGAGCCACAAG	CAL Fluor Red 590
KIMAT1 FISH_9	GGGGCTTGGAGAACGAGT	CAL Fluor Red 590
KIMAT1 FISH_10	CCTGGGTGTACCAGAAGA	CAL Fluor Red 590
KIMAT1 FISH_11	TGTGGAAAGGACCGAGGC	CAL Fluor Red 590
KIMAT1 FISH_12	CCAGGTCAATGAGTACCT	CAL Fluor Red 590
KIMAT1 FISH_13	AGGGTGCCCTTAGTTTTG	CAL Fluor Red 590
KIMAT1 FISH_14	ACAGCTGAAGCCTGTGTT	CAL Fluor Red 590
KIMAT1 FISH_15	AGTGCCCAGGGGTGAATG	CAL Fluor Red 590
KIMAT1 FISH_16	ATACACTCAGGCAGGCTC	CAL Fluor Red 590
KIMAT1 FISH_17	CGCAGTGTGCGATGGGAG	CAL Fluor Red 590
KIMAT1 FISH_18	GATGAGGCCCCAGTTAAA	CAL Fluor Red 590
KIMAT1 FISH_19	CAGACATTCACCTTCCGA	CAL Fluor Red 590
KIMAT1 FISH_20	CAGGTGGTAGGGTTTTCG	CAL Fluor Red 590
KIMAT1 FISH_21	CCTAGCAAAGGGGAGTCA	CAL Fluor Red 590
KIMAT1 FISH_22	AACGGCAGTCAAATTCCT	CAL Fluor Red 590
KIMAT1 FISH_23	GGGGGCGTTGTTTTTGAG	CAL Fluor Red 590
KIMAT1 FISH_24	ATGACCTGTCTTAGCTGC	CAL Fluor Red 590
KIMAT1 FISH_25	CCCTCTCTGAAAACGTAC	CAL Fluor Red 590
KIMAT1 FISH_26	CCGCACTCCTGTATCATT	CAL Fluor Red 590
KIMAT1 FISH_27	GAGATGACACTCTTCCCT	CAL Fluor Red 590

KIMAT1 RAP-MS probes

**Probe ID**	**Sequence**	**Modification**
KIMAT1 Antisense probe	CTTGTTTTCTTCCACTGATG	Biotin
KIMAT1 Antisense probe	AAGGGAGATGGTTTATCTCC	Biotin
KIMAT1 Antisense probe	GGTCAATGAGTACCTCAAGT	Biotin
KIMAT1 Antisense probe	GGTTTTCGAAGGCAGACATT	Biotin
KIMAT1 Antisense probe	ATGGACGATGACCTGTCTTA	Biotin
KIMAT1 Antisense probe	AAAGAGATGACACTCTTCCC	Biotin
UBC Antisense probe 1	TCCATTCAAGACTCGGGAAC	Biotin
UBC Antisense probe 2	GATGGTCTTACCAGTCAGAG	Biotin
UBC Antisense probe 3	AGCTGTTTTCCAGCAAAGAT	Biotin
UBC Antisense probe 4	CTTCACGAAGATTTGCATCC	Biotin

Supplementary Table 3. List of Primers used in this study

**lncRNA cloning in pCDH vector**	
KIMAT1 Fwd	ATA TCTAGA GCCACC ATG TGCTGGATGCTGAGAGG
KIMAT1 Rev	CGC GGATCC CTCCCTTCTGTATCATTTAAAGAG
**lncRNA deletion constructs cloning for *in vitro* transcription**	
KIMAT1	
Deletion contruct 1-4 Fwd	TAA TAC GAC TCA CTA TAG GG TGCTGGATGCTGAG
Deletion contruct 1 Rev	GGCGTCTGTAGGCAGCTTGTGTTAACCAG
Deletion contruct 2 Rev	CCACAAGAAAGACAGGTGGTAGGGTTTTCG
Deletion contruct 3 Rev	CCGCTGACAGGGGGCGTTGTTTTTGAG
Deletion contruct 4 Rev	CTCCCTTCTGTATCATTTAAAGAGATGACACTC
Deletion contruct 5 Fwd	TAA TAC GAC TCA CTA TAG GG CTCCCTTCTGTATCA
Deletion contruct 5 Rev	TGC TGG ATG CTG AGA GGA ACG CAT CAG T
Deletion contruct 6 Fwd	TAA TAC GAC TCA CTA TAG GG GATAAACCATCTCCC
Deletion contruct 7 Fwd	TAA TAC GAC TCA CTA TAG GG ACAAGCTGCCTACA
Deletion contruct 8 Fwd	TAA TAC GAC TCA CTA TAG GG AACCATCAAACTCC
Deletion contruct 6-8 Rev	CTCCCTTCTGTATCATTTAAAGAGATGACACTC
**lncRNA deletion constructs cloning in pCDH vector**	
pCDH KIMAT1 Δ7 Fwd	ATA TCTAGA GCCACC ATG ACAAGCTGCCTACA
pCDH KIMAT1 Δ7 Rev	CGC GGATCC CTCCCTTCTGTATCATTTAAAGAG
pCDH KIMAT1 Δ1 Fwd	ATA TCTAGA GCCACC ATG TGCTGGATGCTGAGAGG
pCDH KIMAT1 Δ1 Rev	CGC GGATCC GGCGTCTGTAGGCAGCTTGTGTTAA
KIMAT1 mut Fwd1	TGCTTCCACTCAATACTCGTTCTCCAAGCCCCCGTGT
KIMAT1 mut Rev1	ACACGGGGGCTTGGAGAACGAGTATTGAGTGGAAGCA
KIMAT1 mut Fwd2	CTTGTGGAACCATCAGGACAGGCAACTGGAGCCTCAG
KIMAT1 mut Rev2	CTGAGGCTCCAGTTGCCTGTCCTGATGGTTCCACAAG
**pri-miRNA *in vitro* processing primers**	
pri-miR-27b Fwd	TAA TAC GAC TCA CTA TAG GG GAGAACAGGTGCATC
pri-miR-27b Rev	AAGTGGTCTCTCATCCTCCAGGATCGCG
**sgRNA primers**	
DHX9 sgRNA Fwd-1	CACC GTTCTCTGTTGTCTCGGTAG
DHX9 sgRNA Rev-1	AAAC CTACCGAGACAACAGAGAAc
DHX9 sgRNA Fwd-2	CACC GTCACACACGGTCCTAAGAG
DHX9 sgRNA Rev-2	AAAC CTCTTAGGACCGTGTGTGAc
NPM1 sgRNA Fwd-1	CACC GCGCAGGACGGCTACGGTAC
NPM1 sgRNA Rev-1	AAAC GTACCGTAGCCGTCCTGCGC
**KIMAT1 promoter cloning in pGL3 basic vector**	
pGL3 basic NP Fwd	ACT GGTACC AGGTCTCCTGAGAGCTCACT
pGL3 basic NP Rev	CAG CTCGAG TATCACTATCACTATCAGGCTAAGC
pGL3 basic KIMAT1 MER101-1 Fwd	ACT GGTACC AAAAGGTCTCTGAAAAGGAATTTGG
pGL3 basic KIMAT1 MER101-1 Rev	TAT CTCGAG CCCCCCACCACTGTTTGAAA

pGL3 basic KIMAT1 MER101-2 Fwd	ACT GGTACC TCTGGCTACAGTTTATCTTGG
pGL3 basic KIMAT1 MER101-2 Rev	TAT CTCGAG TGGTAAACTATGCCCTGGCT
pGL3 basic KIMAT1 MER101-1 BS1 Del F	CCTGGGGTCCCCATTAGGTGCAAATGAAGGATTGAAACGT
pGL3 basic KIMAT1 MER101-1 BS1 Del R	ACGTTTCAATCCTTCATTTGCACCTAATGGGGACCCCAGG
pGL3 basic KIMAT1 MER101-1 BS2 Del F	GCAAATGAAGGATTGGCTTAGTTCTGATTGGTTGG
pGL3 basic KIMAT1 MER101-1 BS2 Del R	CCAACCAATCAGAACTAAGCCAATCCTTCATTTGC

pri-miRNA cloning in pmiRGLO vector

pri-miR-200c Fwd:	TAG CTCGAG TTAAGGCAGTGGGGGGGCAG
pri-miR-200c Rev	CAG TCTAGA CCTTGGGTCAGGCAGCTTCAG
pri-miR-27b Fwd	TAG CTCGAG ACCTCCTGACTTGAGAGTCC
pri-miR-27b Rev	CAG TCTAGA CATCGCTGGGCATAAATAAAGGG
pri-let-7b Fwd	TAA CTCGAG AGCCGCACTGAGAGAGGCGATC
pri-let-7b Rev	TAA TCTAGA CCAGGCCCGGCCTTGCACTGC
pri-miR-17 Fwd	GCC CTCGAG GTTGGGTGATAAAGTAGATATAACCTGAG
pri-miR-17 Rev	CGC TCTAGA TGGTCACAATCTTCAGTTTTACAAGGTG
pri-miR-18a Fwd	GCC CTCGAG ACAAGTATTTGCTAAGTGGAAGC
pri-miR-18a Rev	AGA TCTAGA AAAAGCACTCAACATCAGCAGGCCC

Gene cloning in FLAG-HA-pcDNA 3.1

DHX9 Fwd	CGC GGATCC ATGGGTGACGTTAAAAATTTTCTG
DHX9 Rev	TAC GGTACC TTAATAGCCGCCACCTCCTC
DHX9 dsRBDs Del Fwd	AAGAAGGAAGGAGAGACAGTGG
DHX9 dsRBDs Del Rev	CACTAGTCCAGTGTGGTGGAA
NPM1 DRBD Del Fwd	GGATCCACCGGATCTAGATAACT
NPM1 DRBD Del Rev	AGAACCACCTTTTTCTATACTTGCTTG

KIMAT1 RACE

5'RACE	
KIMAT1 GSP1	CCGCTGACAGGGGGCGTTGTTTTTGAG
KIMAT1 GSP2	ATA CTCGAG CCACAAGAAAGACAGGTG
3'RACE	
KIMAT1 GSP3	ATA GGTACC GATAAACCATCTCCCTTG

Supplementary Table 4. qPCR primers and probes

**qPCR primer**	
KIMAT1 Fwd	AAAACAACGCCCCCTGTC
KIMAT1 Rev	TCCCCTCTCTGAAAACGTACA
CCDST Fwd	GCTGCAGTTCCTGATACTGGT
CCDST Rev	AAATAGTGTTCTCAGGGATCAAAGA
SAMD12-AS1 Fwd	TGGCACCTCTTTACACTGGTT
SAMD12-AS1 Rev	AGGCATCTCACTTGGTGCTC
Human β-actin Fwd	TGACATTAAGGAGAAGCTGTGCTAC
Human β-actin Rev	GAGTTGAAGGTAGTTTCGTGGATG
Mouse β-actin Fwd	TTC ACC ACC ACA GCT GAG AG
Mouse β-actin Rev	ATA GTG ATG ACC TGG CCG TC
UBC Fwd	TAAGGAAGGCATCCCTCCTGAC
UBC Rev	TTCACGAAGATTTGCATCCCAC
DHX9 Fwd	GCAGAAATGACCATTTATATCAAGC
DHX9 Rev	TCCATGTTCTCGTGCAAAAA
NPM1 Fwd	CCGGATGACTGACCAAGAG
NPM1 Rev	TTACAGAAATGAAATAAGACGGAAAA
CDKN1A Fwd	TCACTGTCTTGTACCCTTGTGC
CDKN1A Rev	GGCGTTTGGAGTGGTAGAAA
MYC Fwd	CACCAGCAGCGACTCTGA
MYC Rev	GATCCAGACTCTGACCTTTTGC
TFAP4 Fwd	GCAGGCAATCCAGCACAT
TFAP4 Rev	GGAGGCGGTGTCAGAGGT
RAPGEF4 Fwd	CGAGCAGAAGGACTTCAAGG
RAPGEF4 Rev	AGAGCCCGTTTCCATAACAC
MAP7 Fwd	GACTCCTGACGCCCACAC
MAP7 Rev	AACGAGGACAAATGGGGATA
CCND2 Fwd	GCTGTGCATTTACACCGACA
CCND2 Rev	AGCTGCCAGGTTCCACTTC
RELN Fwd	TCATGTTGATGTGTCCGTGA
RELN Rev	TTTGTGCTTAAATTTCATTTTTCTGT
KRAS Fwd	TTGTGGACGAATATGATCCAAC
KRAS Rev	TCCCTCATTGCACTGTACTCC
DIABLO Fwd	TGACTGCAGTTGGTCTTTCAG
DIABLO Rev	GCGGTTATAGAGGCCTGATCT
BCL2L11 Fwd	CAGGCCTTCAACCACTATCTC
BCL2L11 Rev	AACTCTTGGGCGATCCATATC
BMF Fwd	ACTTCAGCTCTTCCCTCTCA
BMF Rev	GAGTCTGGGTAGCTTTGTCTT
DAP Fwd	CCCGAAGGGAAACTAGAGACT
DAP Rev	GTCTCCTGTATGTGGGTGTTT
pri-miR-200a Fwd	GGATTAGGACGCTCAGGTGT
pri-miR-200a Rev	AGCCCTCTGTTGGGTCCT
pri-miR-200b Fwd	GCGATGCTGTCCTCAGTG
pri-miR-200b Rev	CTCGCTGGGAAGCTCAGTAG
pri-miR-200c Fwd	GGCTCACCAGGAAGTGTCC
pri-miR-200c Rev	AGGATCCCTGCGGAAAAG
pri-miR-7 Fwd	TGCCTTAATTTTTCTTCTGCTTTC
pri-miR-7 Rev	AATGAGAAGTTTGCTTGGTTAAGG
pri-miR-27a Fwd	CATGGGCCCTCTAGGTATCTC
pri-miR-27a Rev	GGAGCTGGAGCCTAGCTGT
pri-miR-27b Fwd	ATTACCACGCAACCACGAC
pri-miR-27b Rev	GCACCTGTTCTCCAATCTGC
pri-miR-139 Fwd	TAGAAGCTGGGACTGGCTTG
pri-miR-139 Rev	CCCACACTGTTGGCTCCT
pri-let-7b Fwd	GCATACACTGGGTCCCACAT
pri-let-7b Rev	GCCTCAGTTTCCCCAGGTA
pri-miR-17 Fwd	CGTGTCTAAATGGACCTCATATCTT
pri-miR-17 Rev	AAACCATACAAATTCAGCATAATCC

pri-miR-18a Fwd	TTCAACAAGTATTTGCTAAGTGGAAG
pri-miR-18a Rev	GGCAATTTAGTCCATGTGTACCT
pri-miR-375 Fwd	CTCTGCTTCTCGGCTCCTC
pri-miR-375 Rev	CCTCTGGAACAACACCAGATG
pri-miR-10b Fwd	TGTTCGCCTGCTTGGTAAC
pri-miR-10b Rev	GCGACAATTTGAAGCAATGA

pre-miR-200a Fwd	GCATCTTACCGGACAGTGCT
pre-miR-200a Rev	GGGTCACCTTTGAACATCGT
pre-miR-200b Fwd	CCGTGGCCATCTTACTGG
pre-miR-200b Rev	TCCGCCGTCATCATTACC
pre-miR-200c Fwd	CCCTCGTCTTACCCAGCAGTG
pre-miR-200c Rev	CCTCCATCATTACCCGGCAG
pre-miR-7 Fwd	GGATGTTGGCCTAGTTCTGTGTGG
pre-miR-7 Rev	GCCTGTGCCATATGGCAGACTG
pre-miR-27a Fwd	CTGAGGAGCAGGGCTTAGCT
pre-miR-27a Rev	GGCGGAACTTAGCCACTGTGAA
pre-miR-27b Fwd	CTAACAAGGTGCAGAGCTTAGC
pre-miR-27b Rev	CTTCAGGTGCAGAACTTAGCC
pre-miR-139 Fwd	GTGTATTCTACAGTGCACGTGTC
pre-miR-139 Rev	TACTCCAACAGGGCCGCGTC
pre-let-7b Fwd	CGGGGTGAGGTAGTAGGTTG
pre-let-7b Rev	CAGGGAAGGCAGTAGGTTGTAT
pre-miR-17 Fwd	GTCAAAGTGCTTACAGTGCAGG
pre-miR-17 Rev	GTCACCATAATGCTACAAGTGCC
pre-miR-18a Fwd	GGTGCATCTAGTGCAGATAGTG
pre-miR-18a Rev	TGCCAGAAGGAGCACTTAGGGC
pre-miR-375 Fwd	ACGAGCCCCTCGCACAAAC
pre-miR-375 Rev	TCACGCGAGCCGAACGAAC
pre-miR-10b Fwd	CCAGAGGTTGTAACGTTGTCT
pre-miR-10b Rev	GCATCGACCATATATTCCCCTA

mature miRNA qPCR probe

Probe	SOURCE	Catalog number	Assay ID
miR-200a-3p	ThermoFisher	4427975	000 502
miR-200b-3p	ThermoFisher	4427975	002 251
miR-200c-3p	ThermoFisher	4427975	002 300
miR-7-5p	ThermoFisher	4440886	005723-mat
miR-27a-3p	ThermoFisher	4427975	000 408
miR-27b-5p	ThermoFisher	4427975	000 409
miR-139-5p	ThermoFisher	4427975	005364_mat
let-7b-3p	ThermoFisher	4427975	002 404
miR-17-5p	ThermoFisher	4427975	002 308
miR-18a-5p	ThermoFisher	4427975	002 422
miR-375	ThermoFisher	4427975	000 564
miR-10b-5p	ThermoFisher	4427975	002 218
RNU48	ThermoFisher	4427975	001 006
SNOR234	ThermoFisher	4427975	001 234

Chip-qPCR primer

KIMAT1 Chip Fwd	GAAGAAACATCTCTGGGCTG
KIMAT1 Chip Rev	TGCTGCTTCTCACTCTGAC
TFAP4-MYC Chip Fwd	CGTGCGGCCAGCTAAGCAAGG
TFAP4-MYC Chip Rev	CATTGCAGCTGCACGGAGCGG

Supplementary Table 5. List of antibodies used in this study

Western blot

**Antibodies**	**Source**	**Cat number**	**Dilution**
RAS	Cell Signaling Technology	8955	1:1000
DHX9	BETHYL Laboratories	A300-855A	1:1000
NPM1	Abcam	Ab10530	1:1000
MYC	Abcam	ab32072	1:1000
β-actin	Santa Cruz Biotechnology	sc-47778	1:1000
α-tubulin	Cell Signaling Technology	2125	1:1000
GAPDH	Cell Signaling Technology	2118	1:1000
pERKs	Cell Signaling Technology	9101	1:1000
Total ERKs	Abcam	ab184669	1:1000
pAKT	Cell Signaling Technology	4060	1:1000
Total AKT	Cell Signaling Technology	9272	1:1000
c-RAF	Santa Cruz Biotechnology	sc-227	1:1000
p21	Abcam	ab109520	1:1000
Ubiqutin	Proteintech	10201-2-AP	1:1000
Drosha	Abcam	ab183732	1:1000
DDX5	Abcam	ab128928	1:1000

Immunoprecipitation

**Antibodies**	**Source**	**Cat number**	**Dilution**
DHX9	BETHYL Laboratories	A300-855A	2 μg per IP
NPM1	Abcam	Ab10530	2 μg per IP
p21	Abcam	ab109520	2 μg per IP
Drosha	Abcam	ab183732	2 μg per IP
IgG	Cell Signaling Technology	2729s	2 μg per IP

Chip

**Antibodies**	**Source**	**Cat number**	**Dilution**
H3K4me3	Diagenode	C15410003-50	5 μg per ChIp
H3K27ac	Diagenode	C15200184-50	5 μg per ChIp
IgG	Abcam	ab171870	5 μg per ChIp
MYC	Cell Signaling Technology	9402	5 μg per ChIp

Immunofluorescence

**Antibodies**	**Source**	**Cat number**	**Dilution**
Vimentin (D21H3)	Cell Signaling Technology	5741	1:150
DHX9	BETHYL Laboratories	A300-855A	1:150
NPM1	Abcam	Ab10530	1:150

Immunohistochemistry

**Antibodies**	**Source**	**Cat number**	**Dilution**
KRAS	Abcam	ab180772	1:100
NPM1	Abcam	Ab10530	1:250
DHX9	Abcam	ab26271	1:100
Casp3	Cell Signaling Technology	9662	1:500
Ki67	Abcam	ab15580	1:500

Secondary antibodies

**Antibodies**	**Source**	**Cat number**	**Dilution**
Anti-Rabbit IgG, HRP-linked Antibody	Cell Signaling Technology	7076	1:5000
Anti-Rabbit IgG, HRP-linked Antibody	Amersham	NA934	1:5000
Donkey Anti-Mouse IgG H&L (Alexa Fluor® 488)	Abcam	ab150105	1:500
Donkey Anti-Rabbit IgG H&L (Alexa Fluor® 555)	Abcam	ab150074	1:500

Supplementary Table 6. List of Plasmids used in this study

**Recombinant DNA**	**Source**	**Cat number**
Pgl3 Basic	Promega	E1751
pGL4.51[*luc2*/CMV/Neo] Vector	Promega	E1320
pmiRGLO	Promega	E1330
DHX9	GeneCopoeia	EX-H1793-M02
NPM1 (FLAG-GFP)	Addgene	17578
p21	Origene	RC201765
$KRAS^{WT}$	Shi et al. 1	N/A
$KRAS^{G12D}$	Shi et al. 1	N/A
Gag:pol	Robert Hawkins lab	N/A
VSVG	Robert Hawkins lab	N/A
REV	Robert Hawkins lab	N/A
FLAG-HA-pcDNA3.1	Addgene	52535
WWP-Luc p21/WAF1 promoter	Addgene	16451
pSpCas9(BB)-2A-GFP	Addgene	48138
pSpCas9(BB)-2A-GFP-sgDHX9	This paper	N/A
pSpCas9(BB)-2A-GFP-sgNPM1	This paper	N/A
pCDH-CMV-MCS-EF1-coGFP Lentivector	System Biosciences	CD511B-1

Supplementary References

1. Shi, L. *et al.* KRAS induces lung tumorigenesis through microRNAs modulation. *Cell Death Dis* **9**, 219 (2018).
