# Supplementary material for: A KRAS-responsive long non-coding RNA controls microRNA processing
Source: Nat Commun. 2021 Apr 1;12:2038. doi: 10.1038/s41467-021-22337-3 (PMC8016872; doi:10.1038/s41467-021-22337-3)
Supplement: Supplementary file 3 — Description of Additional Supplementary Files [file 41467_2021_22337_MOESM3_ESM.pdf]

Description of Additional Supplementary Files

File Name: Supplementary Data 1.

Description: KRAS copy number alterations and mutations in LUAD cohorts.

File Name: Supplementary Data 2.

Description: Gene signature of KRAS WT and KRAS G12D RNA-seq dataset by Gene Set Enrichment Analysis.

File Name: Supplementary Data 3.

Description: Proteins binding to KIMAT1 by RNA Antisense Purification with Mass Spectrometry (RAP-MS).

File Name: Supplementary Data 4.

Description: Network analysis indicating the genes belonging to pathways enriched by KIMAT1 were predicted targets of KIMAT1 suppressed microRNAs.
