# Supplementary material for: A KRAS-responsive long non-coding RNA controls microRNA processing
Source: Nat Commun. 2021 Apr 1;12:2038. doi: 10.1038/s41467-021-22337-3 (PMC8016872; doi:10.1038/s41467-021-22337-3)
Supplement: Supplementary file 8 — Reporting Summary [file 41467_2021_22337_MOESM8_ESM.pdf]

nature research

Corresponding author(s): Michela Garofalo

Last updated by author(s): Feb 6, 2021

Reporting Summary

Nature Research wishes to improve the reproducibility of the work that we publish. This form provides structure for consistency and transparency in reporting. For further information on Nature Research policies, see our Editorial Policies and the Editorial Policy Checklist.

Statistics

For all statistical analyses, confirm that the following items are present in the figure legend, table legend, main text, or Methods section.

n/a	Confirmed	
☐	☒	The exact sample size (n) for each experimental group/condition, given as a discrete number and unit of measurement
☐	☒	A statement on whether measurements were taken from distinct samples or whether the same sample was measured repeatedly
☐	☒	The statistical test(s) used AND whether they are one- or two-sided<br>*Only common tests should be described solely by name; describe more complex techniques in the Methods section.*
☒	☐	A description of all covariates tested
☒	☐	A description of any assumptions or corrections, such as tests of normality and adjustment for multiple comparisons
☐	☒	A full description of the statistical parameters including central tendency (e.g. means) or other basic estimates (e.g. regression coefficient) AND variation (e.g. standard deviation) or associated estimates of uncertainty (e.g. confidence intervals)
☐	☒	For null hypothesis testing, the test statistic (e.g. F, t, r) with confidence intervals, effect sizes, degrees of freedom and P value noted<br>*Give P values as exact values whenever suitable.*
☒	☐	For Bayesian analysis, information on the choice of priors and Markov chain Monte Carlo settings
☒	☐	For hierarchical and complex designs, identification of the appropriate level for tests and full reporting of outcomes
☐	☒	Estimates of effect sizes (e.g. Cohen's d, Pearson's r), indicating how they were calculated

Our web collection on statistics for biologists contains articles on many of the points above.

Software and code

Policy information about availability of computer code

Data collection	IVIS Spectrum In Vivo Imaging, NovoExpress Software (version 1.3.0), Incucyte Zoom software, GelCount software (release 1.2.2.1), BIO-RAD Image Lab Software (version 6.1), LightCycler® 96 System (version 1.1).
Data analysis	Excel 2010, GraphPad Prism 8, Image J, SoftMax Pro 6, CPAT (Coding Potential Assessment) v2.0.0, Cytoscape 3.7.0, Gene Set Enrichment Analysis (GSEA) r package (version 1.2), R environment (3.6.1), R package igraph (version 1.2.4.2), StarSearch, ENCODE, QuPath v0.2.0-m2, RSubread package aligner, RSubread package feature counts, Targetscan (relase 7.2), FastQC, FASTX-Toolkit, miRBase database (release 21), JASPAR (8th release), ALGGEN, FANTOM5, Scaffold 4, R Bioconductor package DESeq2 (v1.26.0), Circular binary segmentation (CBS) algorithm, NovoExpress Software (version 1.3.0).

For manuscripts utilizing custom algorithms or software that are central to the research but not yet described in published literature, software must be made available to editors and reviewers. We strongly encourage code deposition in a community repository (e.g. GitHub). See the Nature Research guidelines for submitting code & software for further information.

Data

Policy information about availability of data

All manuscripts must include a data availability statement. This statement should provide the following information, where applicable:

- Accession codes, unique identifiers, or web links for publicly available datasets
- A list of figures that have associated raw data
- A description of any restrictions on data availability

Mass spectrometry, RNA- seq and ChIP-seq data that support the findings of this study have been deposited in the Gene Expression Omnibus, accession code: GSE124631. List of proteins interacting with KIMAT1 by mass spectrometry is provided in Supplementary Table 3. All other data supporting the findings of this study are available from the corresponding author on reasonable request.

Publicly available datasets
CRISPR design tool:http://tools.genome-engineering.org
FastQC: https://www.bioinformatics.babraham.ac.uk/projects/fastqc
Targetscan: http://www.targetscan.org/vert_72/
Emsembl: http://www.ensembl.org/index.html
USCS browser: https://genome.ucsc.edu/
GSEA: http://www.gsea-msigdb.org/gsea/index.jsp
FASTX-Toolkit: http://hannonlab.cshl.edu/fastx_toolkit/
miRbase: http://www.mirbase.org/
mirDeep2: https://github.com/rajewsky-lab/mirdeep2
GDC Data Portal: https://portal.gdc.cancer.gov/
CCLE: https://portals.broadinstitute.org/ccle
CPAT: http://lilab.research.bcm.edu/cpat/
Genotype-Tissue Expression (GTex): https://gtexportal.org/home/
JASPAR: http://jaspar.genereg.net/
ALGGEN: http://alggen.lsi.upc.es/recerca/frame-recerca.html
ENCODE: https://www.encodeproject.org/
Cytoscape: https://cytoscape.org/

Field-specific reporting

Please select the one below that is the best fit for your research. If you are not sure, read the appropriate sections before making your selection.

[x] Life sciences [ ] Behavioural & social sciences [ ] Ecological, evolutionary & environmental sciences

For a reference copy of the document with all sections, see nature.com/documents/nr-reporting-summary-flat.pdf

Life sciences study design

All studies must disclose on these points even when the disclosure is negative.

Sample size	For in vitro experiments, at least three biological replicates were achieved for most of the experiments, except for the RNA-seq and ChIP-seq. Such sample sizes are typical for the in vitro experiments and sufficient for a statistical analysis. For in vivo experiments, 6-8 mice per group were sufficient to achieve 96% power with a confidence of 95%. Sample size was determined based on our previous experience (Cell Death Dis. 2018 Feb 13;9(2):219;EMBO Mol Med. 2020 Jul 7;12(7):e11099),which is sufficient to generate statistically significant results.
Data exclusions	Mice were excluded when they were presenting signs of illness or distress during the course of the experiments.
Replication	All experiments were repeated at least three times unless otherwise stated in the figure legend.
Randomization	All mice were randomized prior to receive any treatment as described. Or other experiments, cells/samples, were randomly assigned to groups to avoid bias.
Blinding	Mice study including group allocation, dosing, treatment and monitoring were performed by colleagues of Biological Resources Unit; IHC and HE were analyzed by independent researchers. For in vitro experiments, no blinding was necessary since experiments were duplicated and triplicate to verify the results.

Reporting for specific materials, systems and methods

We require information from authors about some types of materials, experimental systems and methods used in many studies. Here, indicate whether each material, system or method listed is relevant to your study. If you are not sure if a list item applies to your research, read the appropriate section before selecting a response.

Materials & experimental systems

n/a	Involved in the study	
[ ]	[x]	Antibodies
[ ]	[x]	Eukaryotic cell lines
[x]	[ ]	Palaeontology and archaeology
[ ]	[x]	Animals and other organisms
[x]	[ ]	Human research participants
[x]	[ ]	Clinical data
[x]	[ ]	Dual use research of concern

Methods

n/a	Involved in the study	
[ ]	[x]	ChIP-seq
[ ]	[x]	Flow cytometry
[x]	[ ]	MRI-based neuroimaging

Antibodies

Antibodies used

Western blot: RAS (Cell Signaling Technology, 8955), DHX9 (BETHYL Laboratories, A300-855A), NPM1 (Abcam, Ab10530), MYC (abcam, ab32072), β-actin (Santa Cruz Biotechnology, sc-47778), α-tubulin (Cell Signaling Technology, 2125), GAPDH (Cell Signaling Technology, 2118), pERKs (Cell Signaling Technology, 9101), Total ERKs (Abcam, ab184669), pAKT (Cell Signaling Technology, 4060), Total AKT (Cell Signaling Technology, 9272), c-RAF (Santa Cruz Biotechnology, sc-227), Ubiquitin(Proteintech, 10201-2-AP), p21 (Abcam, ab109520), Drosha (Abcam, ab183732), DDX5 (Abcam, ab128928). All antibodies were used at 1:1000 dilution.

Immunoprecipitation: DHX9 (BETHYL Laboratories, A300-855A), NPM1 (Abcam, Ab10530), p21 (Abcam, ab109520), Drosha (Abcam, ab183732), IgG (Cell Signaling Technology, 2729s). 2 μg of antibodies were used in each Immunoprecipitation.

ChIP: H3K4me3 (Diagenode, C15410003-50), H3K27ac (Diagenode, C15200184-50), IgG (Abcam, ab171870), MYC (Cell Signaling Technology, 9402).

Immunofluorescence: Vimentin (D21H3) (Cell Signaling Technology, 5741), DHX9 (BETHYL Laboratories, A300-855A), NPM1 (Abcam, Ab10530). All antibodies were used at 1:150 dilution.

Immunohistochemistry: KRAS (Abcam, ab180772,1:100), NPM1 (Abcam, Ab10530, 1:250), DHX9 (Abcam, ab26271,1:100),Ki67 (Abcam, ab15580, 1:500), Casp3 (Cell Signaling Technology, 9662).

Secondary antibodies: Anti-Rabbit IgG HRP-linked Antibody (Cell Signaling Technology, 7076, 1:5000), Anti-Rabbit IgG HRP-linked Antibody (Amersham, NA934, 1:5000), Donkey Anti-Mouse IgG H&L (Alexa Fluor® 488) (Abcam, ab150105, 1:500), Donkey Anti-Rabbit IgG H&L (Alexa Fluor® 555) (Abcam, ab150074,1:500).

This data is available in Supplementary Table 7.

Validation

All commercial antibodies used in this manuscript were previously validated by the manufacturers. Detailed information including specificity, protocols and corresponding applications were provided from the manufacturers' websites:
Western blot:
RAS (https://www.cellsignal.co.uk/products/primary-antibodies/ras-d2c1-rabbit-mab/8955)
DHX9 (https://www.bethyl.com/product/A300-855A/DHX9+Antibody),
NPM1 (https://www.abcam.com/nucleophosmin-antibody-fc82291-ab10530.html),
MYC (https://www.abcam.com/c-myc-antibody-y69-bsa-and-azide-free-ab168727.html)
β-actin (https://www.scbt.com/p/beta-actin-antibody-c4)
α-tubulin https://www.cellsignal.co.uk/products/primary-antibodies/a-tubulin-11h10-rabbit-mab/2125)
GAPDH (https://www.cellsignal.co.uk/products/primary-antibodies/gapdh-14c10-rabbit-mab/2118)
pERKs (https://www.cellsignal.co.uk/products/primary-antibodies/phospho-p44-42-mapk-erk1-2-thr202-tyr204-antibody/9101)
Total ERKs (https://www.abcam.com/wdr77-antibody-8a10-c10-e8-ab184669.html)
pAKT (https://www.cellsignal.co.uk/products/primary-antibodies/phospho-akt-ser473-d9e-xp-rabbit-mab/4060)
Total AKT (https://www.cellsignal.co.uk/products/primary-antibodies/akt-antibody/9272)
c-RAF (https://www.scbt.com/p/raf-1-antibody-c-20)
Ubiquitin(https://www.ptglab.com/products/ubiquitin-Antibody-10201-2-AP.htm)
p21 (https://www.abcam.com/p21-antibody-epr362-ab109520.html)
Drosha (https://www.abcam.com/drosha-antibody-epr12794-ab183732.html)
DDX5 (https://www.abcam.com/ddx5-antibody-epr7240-ab128928.html)

Immunoprecipitation:
DHX9 (https://www.bethyl.com/product/A300-855A/DHX9+Antibody)
NPM1 (https://www.abcam.com/nucleophosmin-antibody-fc82291-ab10530.html)
p21 (https://www.abcam.com/p21-antibody-epr362-ab109520.html)
Drosha (https://www.abcam.com/drosha-antibody-epr12794-ab183732.html)
IgG (https://www.cellsignal.co.uk/products/primary-antibodies/normal-rabbit-igg/2729)

ChIP:
H3K4me3 (https://www.diagenode.com/en/p/h3k4me3-polyclonal-antibody-premium-50-ug-50-ul)
H3K27ac (https://www.diagenode.com/en/p/h3k27ac-monoclonal-antibody-classic-50-mg-50-ml)
IgG (https://www.abcam.com/rabbit-igg-polyclonal-isotype-control-chip-grade-ab171870.html)
MYC (https://www.cellsignal.co.uk/products/primary-antibodies/c-myc-antibody/9402)

Immunofluorescence:
Vimentin (D21H3) (https://www.cellsignal.co.uk/products/primary-antibodies/vimentin-d21h3-xp-rabbit-mab/5741)
DHX9 (https://www.bethyl.com/product/A300-855A/DHX9+Antibody)
NPM1 (https://www.abcam.com/nucleophosmin-antibody-fc82291-ab10530.html)

Immunohistochemistry:
KRAS (https://www.abcam.com/ras-antibody-ab180772.html)
NPM1 (https://www.abcam.com/nucleophosmin-antibody-fc82291-ab10530.html)
DHX9 (https://www.abcam.com/rna-helicase-a-antibody-ab26271.html)

Ki67 (https://www.abcam.com/ki67-antibody-ab15580.html)
Casp3 (https://www.cellsignal.co.uk/products/primary-antibodies/caspase-3-antibody/9662)

Secondary antibodies
Anti-Rabbit IgG HRP-linked Antibody (https://www.cellsignal.co.uk/products/secondary-antibodies/anti-mouse-igg-hrp-linked-antibody/7076)
Anti-Rabbit IgG HRP-linked Antibody (https://www.cytivalifesciences.com/en/us/shop/protein-analysis/blotting-and-detection/blotting-standards-and-reagents/amersham-ecl-hrp-conjugated-antibodies-p-06260?extcmp=g-se-paid&gclid=CjwKCAiA_eb-BRB2EiwAGBnXXlDE4zwOUgYh_U7isNvWnUmqBFsFMWNv0a2jn3OFUYIJHUWPgi0F-xoCC58QAvD_BwE)
Donkey Anti-Mouse IgG H&L (Alexa Fluor® 488) (https://www.abcam.com/donkey-mouse-igg-hl-alexa-fluor-488-ab150105.html)
Donkey Anti-Rabbit IgG H&L (Alexa Fluor® 555) (https://www.abcam.com/donkey-rabbit-igg-hl-alexa-fluor-555-ab150074.html)

Eukaryotic cell lines

Policy information about cell lines

Cell line source(s)	Lung adenocarcinoma cell line H1299, H460, A549, H2228, H1975, CALU1 and CALU6, lung squamous cell carcinoma cell line H520, lung fibroblasts HEL299, lung bronchial epithelial cell line HBEC3-KT, normal human bronchial epithelium BEAS2B cells, kidney embryonic cells HEK293 were purchased from American Type Culture Collection (ATCC) and cultured as suggested by ATCC's guidelines. CORL-23 cells were purchased from Sigma-Aldrich. Type II pneumocytes cells were a kind gift of Prof. Julian Downward (The Institute of Cancer Research, London).
Authentication	Cell lines were authenticated by the suppliers.
Mycoplasma contamination	All cell lines were routinely tested for mycoplasma. No contaminations were detected.
Commonly misidentified lines (See ICLAC register)	Cell lines used in this study are not listed in the ICLAC register.

Animals and other organisms

Policy information about studies involving animals; ARRIVE guidelines recommended for reporting animal research

Laboratory animals	Female NOD/SCID Gamma (NSG) mice aged 4-6 weeks were purchased from Charles River. Mice were observed for signs of illness or distress during the course of the experiments and body weight was measured twice a week. Animals were euthanized after the appearance of predefined criteria like rapid weight loss (>20%) or weight gain (>20% due to ascites) and labored respiration. After euthanasia mice were analysed for the presence of peritoneal tumors. Animals were housed in groups of 4-6 mice per individually ventilated cage in a 12 h light/dark cycle (07:00-19:00 light. 19:00-7:00 dark), with controlled room temperature (23 ± 2°C) and relative humidity (40-50%).
Wild animals	The study did not involve wild animals.
Field-collected samples	No field-collected samples were used in this study.
Ethics oversight	Animal experimental procedures were approved by Cancer Research UK Manchester Institute's Animal Welfare and Ethical Review body in accordance with the Animals Scientific Procedures Act 1986 and according to the ARRIVE guidelines and the Committee of the National Cancer Research Institute guidelines.

Note that full information on the approval of the study protocol must also be provided in the manuscript.

ChIP-seq

Data deposition

- [x] Confirm that both raw and final processed data have been deposited in a public database such as GEO.
- [x] Confirm that you have deposited or provided access to graph files (e.g. BED files) for the called peaks.

Data access links *May remain private before publication.*	https://www.ncbi.nlm.nih.gov/geo/query/acc.cgi?acc=GSE124630
Files in database submission	MG13_2_H1299_H3K4me3_S4_broad_macs2_peaks.broadPeak.filtered.bed<br>MG13_3_H1299_H3K27ac_S5_broad_macs2_peaks.broadPeak.filtered.bed
Genome browser session (e.g. UCSC)	https://genome.ucsc.edu/cgi-bin/hgTracks?db=hg19&lastVirtModeType=default&lastVirtModeExtraState=&virtModeType=default&virtMode=0&nonVirtPosition=&position=chr21%3A33031597%2D33041570&hgsid=740402221_euaWVFM096AP31GTejoDPeaTnNM7

Methodology

Replicates	ChIP-seq experiments were not performed in replicates. Results were validated using data available in ENCODE and by ChIP-qPCR.

Sequencing depth	Paired-end 76 bp reads<br>sample, total, uniquely aligned<br>H1299_Input , 81.23 million reads, 59.92 million reads (73.7%)<br>H1299_H3K4me3 97.18 million reads, 75.82 million reads (78.02%)<br>H1299_H3K27ac 88.44 million reads, 64.65 million reads (73.12%)
Antibodies	H3K4me3 (Diagenode, C15410003-50), H3K27ac (Diagenode, C15200184-50), IgG (Abcam, ab171870), MYC (Cell Signaling Technology, 9402). 5 µg of each antibodies were used in each experiment.
Peak calling parameters	MACS version 2.1.0<br>--broad -g hs --broad-cutoff 0.1 --nomodel --extsize 155
Data quality	Peaks passing the criteria, with a q-value <=0.05 and fold change of 3 over Input were considered to be significant. With these cutoffs we estimated the number of peaks in each sample as follows:<br>Sample, number of peaks<br>MG13_2_H1299_H3K4me3_S4_broad_macs2_peaks.broadPeak.filtered.bed, 22616<br>MG13_3_H1299_H3K27ac_S5_broad_macs2_peaks.broadPeak.filtered.bed, 6016
Software	Bowtie2 was used to align reads to hg19 genome. MACS version 2.1.0 was used to call significant peaks against Input.

Flow Cytometry

Plots

Confirm that:

- [x] The axis labels state the marker and fluorochrome used (e.g. CD4-FITC).
- [x] The axis scales are clearly visible. Include numbers along axes only for bottom left plot of group (a 'group' is an analysis of identical markers).
- [x] All plots are contour plots with outliers or pseudocolor plots.
- [x] A numerical value for number of cells or percentage (with statistics) is provided.

Methodology

Sample preparation	Apoptosis was examined in multiple cell lines using Annexin V assay (Trevigen, 4830-01-K) according to the manufacturer's instructions. Generally, cells were grown in 6-well plates, transfected with GapmeRs or p21 for 48h and then washed with cold PBS and harvested with trypsin. Cell pellets were incubated with Annexin V for 15 minutes in the dark at room temperature. 400 µl 1 x binding buffer was then added to the cells and the percentage of apoptotic cells analyzed using Flow Cytometry (NovoCyte).
Instrument	Flow Cytometry (NovoCyte)
Software	NovoExpress Software (version 1.3.0)
Cell population abundance	Purity was determined by relevant staining using flow cytometry.
Gating strategy	Cells were FSC-A and SSC-A gated. Unstained cells or boiled cells (65oC for 2 minutes) were used as negative and positive control.

- [x] Tick this box to confirm that a figure exemplifying the gating strategy is provided in the Supplementary Information.
